# Therapeutic Effects of Gallic Acid and Alpha‐Tocopherol on Adenine‐Induced Chronic Kidney Disease in Male Wistar Rats

**DOI:** 10.1155/bri/9220531

**Published:** 2026-01-20

**Authors:** Momita Rani Baro, Manas Das, Kishore Sarma, Leena Das, Aashis Dutta, Ananya Chetia, Pliza Kalita

**Affiliations:** ^1^ Department of Zoology, Animal Physiology and Biochemistry Laboratory, Gauhati University, Guwahati, 781014, Assam, India, gauhati.ac.in; ^2^ Department of Computational Biology and Biotechnology, Mahapurusha Srimanta Sankaradeva Viswavidyalaya, Rupnagar, Guwahati, 781032, Assam, India

**Keywords:** adenine diet, alpha-tocopherol, chronic kidney disease, gallic acid, natural compound

## Abstract

Chronic kidney disease (CKD) is a major health issue associated with oxidative stress and inflammation that leads to progressive renal damage. Natural antioxidants, gallic acid (GA) and alpha‐tocopherol (AT), have gained attention for their strong free radical‐scavenging, inflammation‐reducing, and tissue‐repairing properties, and their individual or combined administration may offer therapeutic potential in CKD management. This experiment was designed to explore the potential ameliorative effects of GA and AT against CKD induced by adenine in male rats. Adult rats weighing 180–220 g (*n* = 48) were distributed among eight experimental groups. Except for Group I (control), all groups received a standard rat diet supplemented with 0.75% (w/w) adenine for 4 weeks to induce CKD. During the same period, the experimental groups received oral treatments of GA and AT at doses of 100 and 400 mg/kg body weight, respectively, as well as their combinations (GA–AT) at the same doses. The treatments were administered simultaneously for 4 weeks to evaluate their effects on adenine‐induced CKD. The results indicated that both GA and the combination of GA–AT were significantly more effective than AT alone in improving renal function markers such as uric acid, creatinine, albumin, and urea. Additionally, these treatments led to better outcomes for serum concentrations of these markers and oxidative stress biomarkers. Histopathological analysis confirmed the beneficial effects on kidney tissue compared to the diseased group. Moreover, both GA and the GA–AT combination treatments showed superior results in the relative expression of mRNA markers related to kidney function, including Igfbp7, Vcam1, and Timp2. Molecular docking studies demonstrated notable binding affinities and interactions between key kidney markers and selected GA and AT compounds. These findings suggest that GA, particularly in combination with AT, effectively restores kidney function in adenine‐induced CKD, supporting further research to optimize their clinical applications in CKD management.

## 1. Introduction

In recent years, natural compounds have garnered significant interest for their therapeutic potential in managing various diseases. Many of these plant‐derived molecules can fight oxidative stress, reduce inflammation, and protect against infections, which makes them interesting candidates for both prevention and treatment. Their multiple modes of action also suggest they could offer safer and more effective alternatives to some conventional synthetic drugs. One such disease where compounds may have particular relevance is chronic kidney disease (CKD). CKD is marked by a gradual loss of kidney function, resulting in the buildup of metabolic waste and disturbances in electrolyte balance within the body [1]. The clinical and societal burden of CKD is substantial globally, with increasing prevalence and limited effective treatment options [2]. CKD arises from multiple interrelated processes, including oxidative stress, inflammation, fibrotic changes, and vascular endothelial damage, which collectively lead to progressive nephron loss and a decline in renal function [1, 2]. Oxidative stress is a significant factor in the intricate biological processes of CKD, contributing to its initiation and continued progression. Increased production of reactive oxygen species (ROS) and impaired antioxidant defenses contribute to cellular damage and apoptosis within the kidneys, exacerbating disease progression [3]. Furthermore, chronic inflammation and dysregulation of immune responses further perpetuate kidney injury, underscoring the necessity for therapeutic approaches that effectively manage oxidative stress and inflammation during CKD treatment [4]. Despite available treatments, disease progression frequently continues, highlighting the need for safer and more effective complementary approaches.

In this context, gallic acid (GA) and alpha‐tocopherol (AT) have attracted considerable interest as potential therapeutic agents due to their capacity to alleviate oxidative stress and inflammation, both of which play a pivotal role in the progression of CKD. GA is a polyphenolic compound, known chemically as 3, 4, 5‐trihydroxybenzoic acid and recognized for its significant antioxidant and notable pharmacological potential. Found abundantly in fruits such as grapes and pomegranates, as well as in gallnuts and tea leaves, GA offers a broad spectrum of health benefits. Studies have highlighted GA’s potent antioxidant activity, which helps neutralize harmful free radicals and minimize oxidative stress. In addition, it displays significant inflammation‐reducing effects and shows promise as an anticancer agent by inhibiting the growth of cancer cells. GA also possesses antimicrobial properties, including antifungal and antiviral activity, offering protection against a range of pathogens. Beyond these effects, it supports liver and brain health through hepatoprotective and neuroprotective actions. Moreover, GA contributes to tissue repair and regeneration, particularly aiding recovery in the liver and kidneys. Altogether, these varied therapeutic properties position GA as a promising compound for use in both prevention and therapeutic applications [5–7]. On the other side, AT is one of the eight forms of vitamin E, a lipid‐soluble molecule renowned for its role as a chain‐breaking antioxidant and its inflammation‐reducing properties. Studies have shown that AT is effective in preventing and treating various health conditions, including kidney function deterioration, cardiovascular complications, and cancer. Additionally, it helps protect renal tissues from oxidative damage, underscoring its therapeutic potential in managing and mitigating oxidative stress‐related diseases [8–10]. It is renowned for its potent antioxidant properties and ability to protect against lipid peroxidation and also plays a crucial role in maintaining cellular membrane integrity and mitigating oxidative stress by neutralizing ROS [11]. This antioxidant capability and its ability to protect cell membranes and regulate inflammatory responses underscore AT’s potential as a promising therapeutic agent in diseases where oxidative stress is a significant contributor, such as CKD.

Preclinical studies have provided compelling evidence supporting the efficacy of GA and AT in mitigating kidney injury and preserving renal function in various CKD models. For instance, treatment with GA has been shown to attenuate oxidative stress markers, reduce inflammatory cytokine levels, and improve renal histopathology in animal models induced by nephrotoxic agents [12, 13]. Similarly, AT supplementation has been associated with decreased oxidative stress and improved renal function in experimental models of diabetic nephropathy and other CKD conditions [11]. In addition to experimental evidence, clinical studies exploring the therapeutic potential of GA and AT in CKD are emerging. Although limited in number, these studies have reported promising outcomes, including improvements in markers of kidney function, reduction in proteinuria, and attenuation of oxidative stress biomarkers in kidney nephrotoxic patients [13–15]. However, further well‐structured clinical studies with good design are necessary to evaluate the safety, effectiveness, and ideal dosing of GA and AT in CKD management. Molecular docking studies have provided mechanistic insights into the interactions of GA and AT with key biomolecules involved in renal pathophysiology. These studies have revealed favorable binding affinities and potential interactions with oxidative stress markers, inflammatory mediators, and signaling pathways critical for kidney function and homeostasis [11, 16].

In conclusion, GA and AT might represent promising natural compounds for the management of CKD due to their potent antioxidant, anti‐inflammatory, and renoprotective properties. Their ability to target multiple pathways implicated in CKD pathogenesis makes them attractive candidates for future therapeutic strategies. Further research, especially clinical trials and studies on their mechanisms, is needed to better understand their capability, and based on these considerations, the present study was designed to investigate their protective effects and evaluate their possible role in CKD management.

## 2. Study Design and Methodology

### 2.1. Reagents and Kits

Adenine, GA, AT, olive oil, and kits for the estimation of nitric oxide (NO) (EZAssay) were procured from HiMedia. Urea, uric acid, creatinine, albumin, phosphorus, and the liver enzyme alkaline phosphatase (ALP), alanine aminotransferase (ALT), and aspartate aminotransferase (AST) kits were obtained from Coral Clinical Systems (India). RNA isolation reagent (RNAiso Plus), a kit for synthesis of cDNA, and TB Green Premix Ex Taq were sourced from Takara Bio Inc. (Japan). Analytical‐grade chemicals and reagents were used for all experimental procedures.

### 2.2. Animal Details

Eight‐week‐old Wistar rats (male; 180–220 g) were chosen for the study and adapted to laboratory conditions for a minimum of two weeks before experimentation. Animals were housed in solid‐bottomed propylene cages with sawdust bedding, given food and water ad libitum, and kept within a controlled environment with a temperature range of 27°C–30°C, ambient humidity of 75%–87%, and a 12‐h photoperiod [17]. All study procedures received approval from the Institutional Animal Ethics Committee (IAEC/Per/2019/PP‐IAEC/2022‐4) and followed guidelines of the Animal Research: Reporting of In Vivo Experiments (ARRIVE) and Committee for the Control and Supervision of Experiments on Animals (CCSEA).

### 2.3. Animal Study Outline

Healthy adult male rats (Wistar) were categorized into eight different experimental sets in a random manner, with six animals (*n* = 6, per group) in each group (Table 1). To induce CKD, a standard diet mixed with adenine (0.75% [w/w]; 320 mg/kg bw per day) was fed to the animals continuously across 28 days [18].

**Table 1:** Experimental groups and dosage regimen of adenine, gallic acid, and alpha‐tocopherol during the treatment period.

Experimental groups	Treatment	Doses		
		Adenine‐induced	Gallic acid	Alpha‐tocopherol
Group I	Control (C)	—	—	—
Group II	Adenine‐induced (AI)	320 mg/kg bw	—	—
Group III	Adenine + gallic acid low dose (A + GALD)	320 mg/kg bw	100 mg/kg bw	—
Group IV	Adenine + gallic acid high dose (A + GAHD)	320 mg/kg bw	400 mg/kg bw	—
Group V	Adenine + alpha‐tocopherol low dose (A + ATLD)	320 mg/kg bw	—	100 mg/kg bw
Group VI	Adenine + alpha‐tocopherol high dose (A + ATHD)	320 mg/kg bw	—	400 mg/kg bw
Group VII	Adenine + gallic acid low dose + alpha‐tocopherol low dose (A + GALD + ATLD)	320 mg/kg bw	100 mg/kg bw	100 mg/kg bw
Group VIII	Adenine + gallic acid high dose + alpha‐tocopherol high dose (A + GAHD + ATHD)	320 mg/kg bw	400 mg/kg bw	400 mg/kg bw

During the same period, GA and AT were administered as treatments. GA after being dissolved in distilled water was administered orally at two doses: 100 mg/kg body weight (low) and 400 mg/kg body weight (high). AT was dissolved in olive oil and administered orally at 100 mg/kg body weight (low) and 400 mg/kg body weight (high) doses over a period of 28 days [6, 19–21]. Furthermore, the combined GA–AT treatment was also given orally at low (100 mg/kg body weight) and high (400 mg/kg body weight) doses for the same duration.

The experimental groups were as follows:

1. Group I (C): Received no treatments, control set.
2. Group II (AI): Received adenine (320 mg/kg bw) to induce CKD, without any treatment.
3. Group III (A + GALD): Received adenine and a low dose of GA (100 mg/kg bw).
4. Group IV (A + GAHD): Received adenine and a high dose of GA (400 mg/kg bw).
5. Group V (A + ATLD): Received adenine and a low dose of AT (100 mg/kg bw).
6. Group VI (A + ATHD): Received adenine and a high dose of AT (400 mg/kg bw).
7. Group VII (A + GALD + ATLD): Received adenine along with low doses of both GA and AT at 100 mg/kg body weight.
8. Group VIII (A + GAHD + ATHD): Received adenine with high doses of both GA and AT at 400 mg/kg body weight.

After completing the 28‐day treatment period, anesthesia was administered intraperitoneally using ketamine (50–75 mg/kg) combined with xylazine (10 mg/kg) [22], and once the animals were fully anesthetized, blood was drawn using a 1‐mL syringe through cardiac puncture. Blood samples (approximately 2000 μL) were collected from the animals, and the serum was separated by centrifugation (Eppendorf MiniSpin Centrifuge) at 5000 rpm for 15 min and stored at −20°C in microcentrifuge tubes for further analysis. Then, the animals were prepared for dissection where the abdominal cavity was carefully opened, and both kidneys were removed under sterile conditions. One set of kidneys from each group was preserved at −20°C for further investigation, whereas another set was fixed for histopathological analysis.

### 2.4. Assessment of Body Weight

During the treatment period, the assessment of animals’ body weight was carried out weekly and recorded to examine any changes [23].

### 2.5. Collection and Analysis of Urine Samples

For the evaluation of urine output and water intake, the animals were kept in urine collection cages individually for collecting the 24‐h samples the day before the end of the treatment [24]. The collected samples of urine were then kept at −20°C and processed for urine analysis. The analysis of creatinine, uric acid, urea, and albumin in urine samples was examined following the manufacturer’s protocol using commercially available diagnostic kits.

### 2.6. Evaluation of Kidney Weight Relative to Body Weight

The relative kidney weight was obtained by comparing the recorded left kidney weight of all animals of each group with the corresponding terminal body weight at the end of the study [25].

### 2.7. Serum Biochemical Parameters

Serum biochemical parameters, including urea, albumin, NO, creatinine, phosphorus, uric acid, and liver‐associated markers, ALP, AST, and ALT, were estimated in all experimental sets using specific assay kits, following the procedures provided with each kit. The concentration of blood urea nitrogen (BUN) was derived from the serum urea measurements.

### 2.8. Determination of Enzymatic Oxidative Stress Biomarkers

Tissues from the kidney of each experimental set were carefully excised and promptly rinsed with 0.9% sodium chloride solution to remove any residual blood. Each sample was processed in an ice‐cold phosphate buffer solution and centrifuged at 10, 000 × *g* for 15 min at a temperature of 4°C. The obtained supernatant was then carefully separated and used for further analysis of antioxidant parameters, including enzymatic (SOD, CAT, and GST) and nonenzymatic (GSH and MDA) markers [24]. The total protein concentration was quantified using the Lowry technique, with bovine serum albumin (BSA) serving as the reference sample [26]. The enzymatic activities of SOD, CAT, and GST were evaluated using previously established protocols [27–29], whereas GSH activity was determined by the established Ellman‐based procedure [30]. The extent of lipid oxidative damage was estimated by measuring MDA production as described earlier [31].

### 2.9. Histopathology

Histopathological examinations of the kidneys were conducted to study changes in both the control and treated groups following a standard histological protocol. The tissue samples were preserved in a neutral‐buffered 10% formaldehyde solution, dehydrated through a graded series of alcohol, treated with xylene for tissue transparency, and then processed and cast in paraffin blocks. Thin tissue sections measuring 5 μm in thickness were obtained through microtomy. Hematoxylin and eosin were applied to the tissue sections for histological staining, sealed with DPX mounting medium, and subsequently examined under a light microscope. Representative photomicrographs were captured for detailed evaluation [32].

### 2.10. Gene Expression Profiling via qPCR

Kidney tissues from all experimental sets were processed for RNA isolation using RNAiso Plus reagent, employing the established chloroform–isopropanol extraction procedures [33]. The RNA samples were purified from genomic DNA by DNase I treatment under RNase‐free conditions. RNA concentration and purity were determined fluorometrically using the Qubit 3.0 system, and the integrity and purity of the isolated RNA were evaluated using 1% agarose gel electrophoresis. Subsequently, cDNA was generated from 0.5 μg of total RNA from each sample using a first‐strand synthesis kit as per the instructions provided with the kit.

Quantitative PCR assessment was carried out using reverse‐transcribed cDNA and specific primer sets (Table S1) on a Qiagen Rotor‐Gene Q system. The relative expression levels of target genes were normalized to the housekeeping gene *Gapdh* for all analyzed samples. Amplification reactions were carried out using TB Green Premix Ex Taq reagent, following the manufacturer’s instructions, with each mixture containing appropriate concentrations of master mix, primers, reference dye, nuclease‐free sterile water, and the synthesized cDNA sample. The amplification procedure included an initial denaturation step, followed by multiple cycles of denaturation, primer annealing at optimized temperatures, and extension under standardized conditions, as described previously [34]. Relative quantification of mRNA abundance was estimated following the 2^−ΔΔCt^ comparative cycle threshold procedure [35, 36].

### 2.11. Molinspiration

In the present study, the Molinspiration software was employed to determine and assess the molecular properties, as well as predict the bioactivity scores of GA and AT compounds, following Lipinski’s rule of five criteria [37]. The ligands, GA and AT, were acquired via the ChemSpider platform in both SMILES and .mol file formats.

### 2.12. In Silico Interaction Analysis

In silico docking analysis was employed to examine potential binding interaction patterns of GA and AT toward the selected protein targets (Tnf, Tlr4, Igfbp7, Kl, Clu, Timp1, Vcam1, Timp2, Apoe, and Cystatin C). The 3D conformer structures of the ligands in SDF format were obtained from the PubChem repository (Table S2) and followed by conversion to PDB format with Open Babel. Rat protein 3D structures were obtained from the AlphaFold database, followed by binding site prediction with the CASTp [38] server (Table S3).

The AutoDockTools (ADT) program (4.2.6) was employed in the preparation of proteins and ligands for molecular docking evaluation [39]. The Lamarckian genetic algorithm (LGA) was used to individually dock GA and AT with the selected key proteins. Interactions of the residues of the amino acids of Tnf, Tlr4, Igfbp7, Kl, Clu, Timp1, Vcam1, Timp2, Apoe, and Cystatin C with ligands (GA and AT) were examined using Discovery Studio 2021.

### 2.13. Statistical Data Evaluation

Experiments were independently repeated three times for accuracy, and the data are reported as mean values together with the standard error. All the analyses were carried out with SPSS version 16 and the statistical comparisons of all values were conducted using one‐way analysis of variance (ANOVA), followed by Tukey’s multiple range test to determine significant differences. A probability score < 0.05 was deemed as the threshold of statistical significance for all analyses.

## 3. Results

### 3.1. Body Weight and Relative Kidney Weight

**Figure 1:**
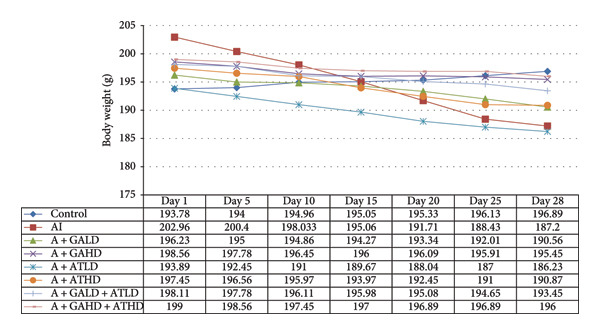
Graph illustrating the variation in body weights of rats across all experimental groups: control, adenine‐induced (AI), adenine + gallic acid low dose (A + GALD), adenine + gallic acid high dose (A + GAHD), adenine + alpha‐tocopherol low dose (A + ATLD), adenine + alpha‐tocopherol high dose (A + ATHD), adenine + gallic acid low dose + alpha‐tocopherol low dose (A + GALD + ATLD), and adenine + gallic acid high dose + alpha‐tocopherol high dose (A + GAHD + ATHD). Each group consisted of six animals (*n* = 6). Chronic kidney disease (CKD) was induced using adenine (320 mg/kg, p.o.), and treatments were administered orally as follows: GALD (100 mg/kg), GAHD (400 mg/kg), ATLD (100 mg/kg), ATHD (400 mg/kg), GALD + ATLD (100 mg/kg), and GAHD + ATHD (400 mg/kg).

**Figure 2:**
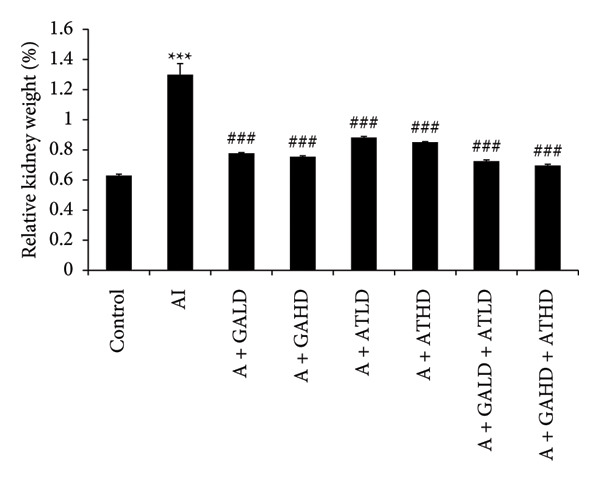
Graph illustrating the relative kidney weight (%) of rats across different experimental groups: control, adenine‐induced (AI), adenine + gallic acid low dose (A + GALD), adenine + gallic acid high dose (A + GAHD), adenine + alpha‐tocopherol low dose (A + ATLD), adenine + alpha‐tocopherol high dose (A + ATHD), adenine + gallic acid low dose + alpha‐tocopherol low dose (A + GALD + ATLD), and adenine + gallic acid high dose + alpha‐tocopherol high dose (A + GAHD + ATHD). Chronic kidney disease (CKD) was induced by oral administration of adenine (320 mg/kg, p.o.), and treatments were administered orally as follows: GALD (100 mg/kg), GAHD (400 mg/kg), ATLD (100 mg/kg), ATHD (400 mg/kg), GALD + ATLD (100 mg/kg), and GAHD + ATHD (400 mg/kg). Data are expressed as mean ± SEM. Statistical significance: ^∗^ *p* < 0.05, ^∗∗^ *p* < 0.01, and ^∗∗∗^ *p* < 0.001 vs. control group and ^#^ *p* < 0.05, ^##^ *p* < 0.01, and ^###^ *p* < 0.001 vs. AI group (one‐way ANOVA).

Throughout the experimental period, rats in the adenine‐induced group showed a steady decline in body weight. This suggests the beginning of chronic kidney dysfunction. In contrast, animals that received GA, AT, or both GA–AT treatments gained weight noticeably compared to the adenine group. The improvement in weight among the treated groups became clearer as the treatment went on. This indicates a protective effect of these compounds against metabolic changes caused by adenine. The control group kept a relatively stable body weight during the study (Figure 1). The relative kidney weight was also significantly higher in the adenine‐induced group than in the control animals, indicating kidney enlargement. In the treatment groups, giving GA, AT, or their combinations, GA–AT, led to a significant drop in relative kidney weight compared to the adenine group. This showed a trend toward normalization over the study period (Figure 2).

### 3.2. Assessment of Urinary Biochemical and Physiological Indicators

Biochemical analysis of urine samples showed that adenine administration caused a significant increase in urea, uric acid, and creatinine levels. It also led to a noticeable increase in albumin concentration compared to the control group. Treatment with GA at both 100 mg/kg and 400 mg/kg doses, as well as the combined administration of GA–AT at the same doses, significantly brought these altered parameters back toward normal values. Among the treatment groups, the combination treatment (GA–AT) at both doses produced the most significant improvement. This suggests a stronger protective effect against adenine‐induced kidney damage (Table 2).

**Table 2:** Analysis of biochemical and physiological markers in urine samples.

Parameters	Control	Adenine‐induced (AI)	Adenine + gallic acid low dose (A + GALD)	Adenine + gallic acid high dose (A + GAHD)	Adenine + alpha‐tocopherol low dose (A + ATLD)	Adenine + alpha‐tocopherol high dose (A + ATHD)	Adenine + gallic acid low dose + alpha‐tocopherol low dose (A + GALD + ATLD)	Adenine + gallic acid high dose + alpha‐tocopherol high dose (A + GAHD + ATHD)
Urea (mg/dL)	13.55 ± 0.05	32.77 ± 0.02^∗^	22.45 ± 0.04^#^	20.67 ± 0.02^#^	29.42 ± 0.03^#^	27.89 ± 0.06^#^	21.85 ± 0.035^#^	19.87 ± 0.02
Uric acid (mg/dL)	5.1 ± 0.05	14.41 ± 0.05^∗∗^	8.85 ± 0.02^#^	7.56 ± 0.06	11.95 ± 0.029^#^	11.3 ± 0.08	8.90 ± 0.08^#^	6.78 ± 0.02^#^
Creatinine (μmol/L)	3.5 ± 0.14	19.09 ± 0.36^∗∗^	5.55 ± 0.74^##^	5.00 ± 0.05^#^	8.36 ± 0.03^#^	6.89 ± 0.98	9.35 ± 0.22^#^	6.78 ± 0.03^#^
Albumin (mmol/L)	11.4 ± 1.20^∗^	25.7 ± 1.20	18.4 ± 1.20^#^	14.78 ± 0.02^#^	20.89 ± 0.87	19.67 ± 0.06	18.8 ± 1.8^#^	13.3 ± 1.4
Water intake (mL/24 h)	24.9 ± 0.50	55.9 ± 1.0^∗^	34.9 ± 0.23^#^	33.87 ± 0.6	40 ± 0.2	39.56 ± 0.02^#^	30.56 ± 0.3	28.2 ± 0.03^#^
Urine output (mL/24 h)	17.4 ± 1.10	45.5 ± 0.56^∗^	32.2 ± 0.20	27.95 ± 0.04^#^	40.2 ± 0.20	38.08 ± 0.03^#^	33.2 ± 0.30	26.78 ± 0.04

*Note:* Values in the table are expressed as mean ± SEM (n = 6). Statistical significance was determined by one‐way ANOVA followed by Tukey′s multiple comparison post‐hoc test. ^∗^ *p* < 0.05, ^∗∗^ *p* < 0.01 vs the control group; ^#^ *p* < 0.05, ^##^ *p* < 0.01 vs the AI group.

### 3.3. Evaluation of Serum Biochemical Profile

Adenine administration resulted in a notable increase in serum urea, creatinine, uric acid, and BUN levels when compared to the control group. Oral treatment with GA at doses of 100 mg/kg and 400 mg/kg, along with the combined administration of GA–AT at the same doses, significantly lowered these elevated serum parameters (Figures 3(a), 3(b), 3(c), and 3(d)). In contrast, the serum albumin concentration, which was significantly lower in the adenine‐induced group, showed a significant improvement after treatment with GA alone or combined with AT. Additionally, adenine exposure caused an increase in NO and phosphorus levels, both of which were effectively brought back to near‐normal levels in the treatment groups receiving GA and GA plus AT (Figures 4(a), 4(b), and 4(c)).

Figure 3Graphs showing the (a) urea, (b) creatinine, (c) uric acid, and (d) BUN activities in serum of the rats of all the experimental groups: control, adenine‐induced (AI), adenine + gallic acid low dose (A + GALD), adenine + gallic acid high dose (A + GAHD), adenine + alpha‐tocopherol low dose (A + ATLD), adenine + alpha‐tocopherol high dose (A + ATHD), adenine + gallic acid low dose + alpha‐tocopherol low dose (A + GALD + ATLD), and adenine + gallic acid high dose + alpha‐tocopherol high dose (A + GAHD + ATHD). Animals (*n* = 6 per group) were given AI (320 mg/kg p.o.) to induce chronic kidney disease (CKD) and GALD (100 mg/kg p.o.), GAHD (400 mg/kg p.o.), ATLD (100 mg/kg p.o.), ATHD (400 mg/kg p.o.), GALD + ATLD (100 mg/kg p.o.), and GAHD + ATHD (400 mg/kg p.o.) orally as treatment. Serum samples were used to evaluate the urea, creatinine, uric acid, and BUN activities. Levels of urea, creatinine, uric acid, and BUN activities in serum are expressed as mean ± SEM (*n* = 6). The values are statistically significant at ^∗^ *p* < 0.05, ^∗∗^ *p* < 0.01, and ^∗∗∗^ *p* < 0.001 vs. the control group and ^#^ *p* < 0.05, ^##^ *p* < 0.01, and ^###^ *p* < 0.001 vs. the AI group, determined by one‐way ANOVA.

**Figure:**
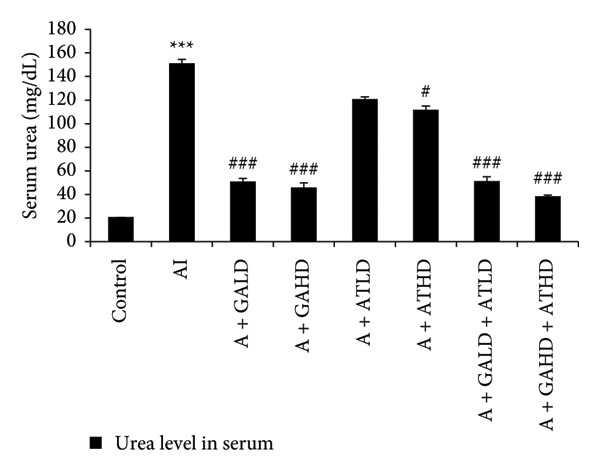
(a)

**Figure:**
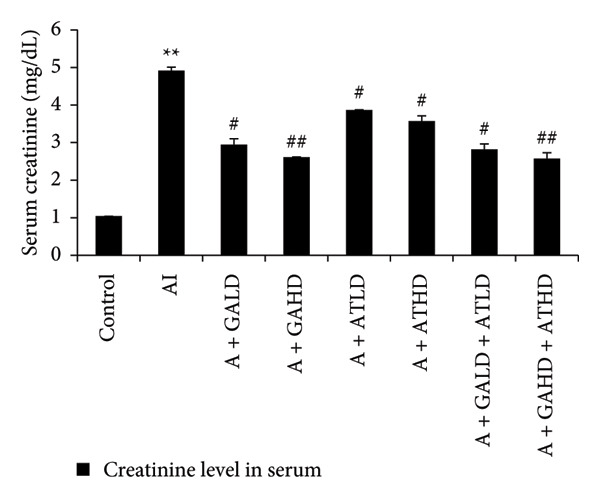
(b)

**Figure:**
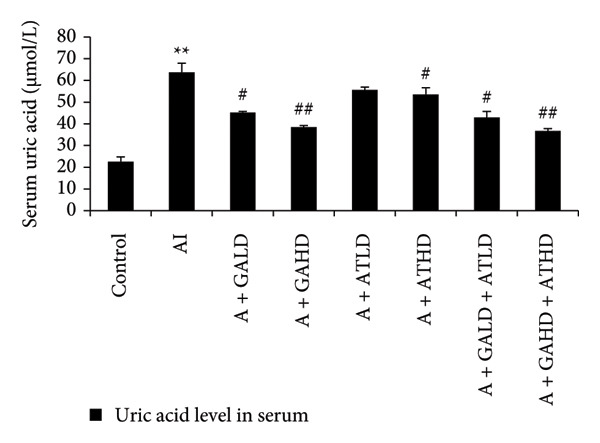
(c)

**Figure:**
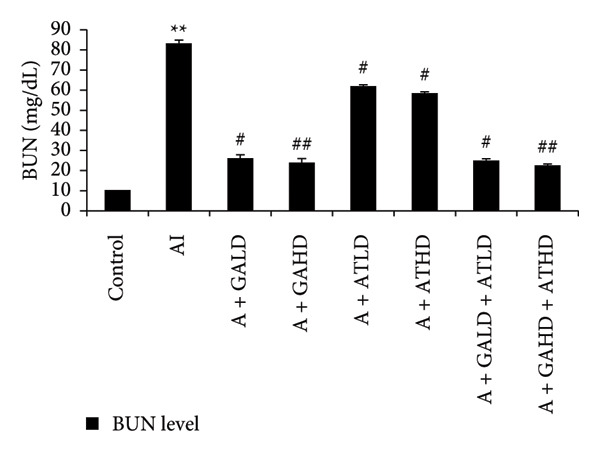
(d)

Figure 4Graphs showing the (a) albumin, (b) NO, and (c) phosphorus activities in serum of the rats of all the experimental groups: control, adenine‐induced (AI), adenine + gallic acid low dose (A + GALD), adenine + gallic acid high dose (A + GAHD), adenine + alpha‐tocopherol low dose (A + ATLD), adenine + alpha‐tocopherol high dose (A + ATHD), adenine + gallic acid low dose + alpha‐tocopherol low dose (A + GALD + ATLD), and adenine + gallic acid high dose + alpha‐tocopherol high dose (A + GAHD + ATHD). Animals (*n* = 6 per group) were given AI (320 mg/kg p.o.) to induce chronic kidney disease (CKD) and GALD (100 mg/kg p.o.), GAHD (400 mg/kg p.o.), ATLD (100 mg/kg p.o.), ATHD (400 mg/kg p.o.), GALD + ATLD (100 mg/kg p.o.), and GAHD + ATHD (400 mg/kg p.o.) orally as treatment. Serum samples were used to evaluate the albumin, NO, and phosphorus activities. Levels of albumin, NO, and phosphorus activities in serum are expressed as mean ± SEM (*n* = 6). The values are statistically significant at ^∗^ *p* < 0.05, ^∗∗^ *p* < 0.01, and ^∗∗∗^ *p* < 0.001 vs. the control group and ^#^ *p* < 0.05, ^##^ *p* < 0.01, and ^###^ *p* < 0.001 vs. the AI group, determined by one‐way ANOVA.

**Figure:**
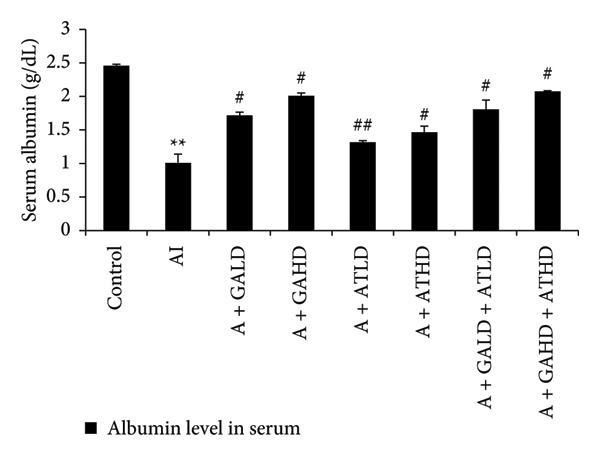
(a)

**Figure:**
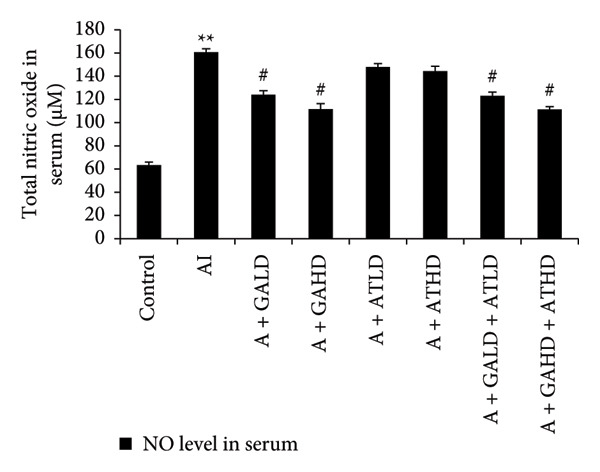
(b)

**Figure:**
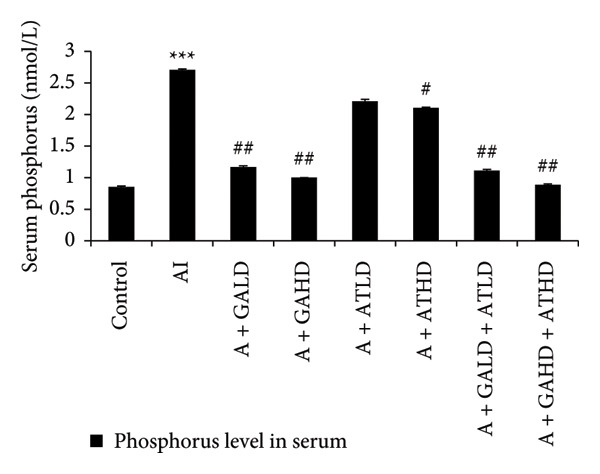
(c)

### 3.4. Assessment of Serum Hepatic Enzyme Activities

Serum biochemical tests of liver enzymes were conducted to check for potential liver damage linked to adenine. Rats with adenine‐induced CKD showed a significant increase in AST, ALT, and ALP levels compared to the control group. This rise indicates liver stress possibly due to kidney problems. Treatment with GA, AT, and their combination significantly lowered these enzyme levels compared to the adenine group. In the treated groups, GA at both 100 mg/kg and 400 mg/kg doses, along with its combination with AT at the same doses, led to a noticeable drop in AST, ALT, and ALP levels, showing marked improvement in liver function (Figure 5(a), 5(b), and 5(c)).

Figure 5Graphs showing the (a) ALT, (b) AST, and (c) ALP activities in serum of the rats of all the experimental groups: control, adenine‐induced (AI), adenine + gallic acid low dose (A + GALD), adenine + gallic acid high dose (A + GAHD), adenine + alpha‐tocopherol low dose (A + ATLD), adenine + alpha‐tocopherol high dose (A + ATHD), adenine + gallic acid low dose + alpha‐tocopherol low dose (A + GALD + ATLD), and adenine + gallic acid high dose + alpha‐tocopherol high dose (A + GAHD + ATHD). Animals (*n* = 6 per group) were given AI (320 mg/kg p.o.) to induce chronic kidney disease (CKD) and GALD (100 mg/kg p.o.), GAHD (400 mg/kg p.o.), ATLD (100 mg/kg p.o.), ATHD (400 mg/kg p.o.), GALD + ATLD (100 mg/kg p.o.), and GAHD + ATHD (400 mg/kg p.o.) orally as treatment. Serum samples were used to evaluate the ALT, AST, and ALP activities. Levels of ALT, AST, and ALP activities in serum are expressed as mean ± SEM (*n* = 6). The values are statistically significant at ^∗^ *p* < 0.05, ^∗∗^ *p* < 0.01, and ^∗∗∗^ *p* < 0.001 vs. the control group and ^#^ *p* < 0.05, ^##^ *p* < 0.01, and ^###^ *p* < 0.001 vs. the AI group, determined by one‐way ANOVA.

**Figure:**
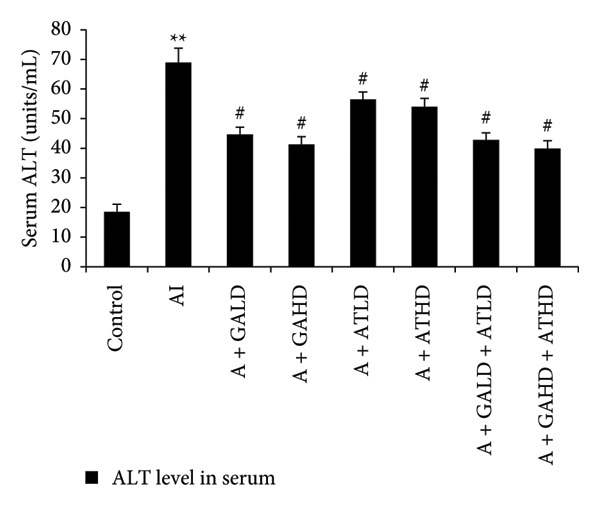
(a)

**Figure:**
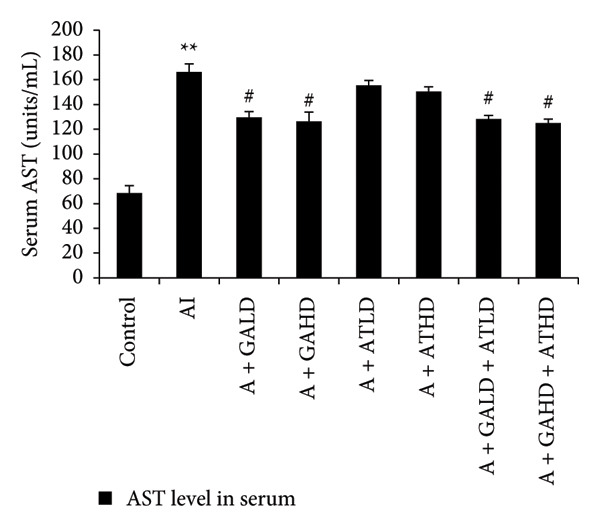
(b)

**Figure:**
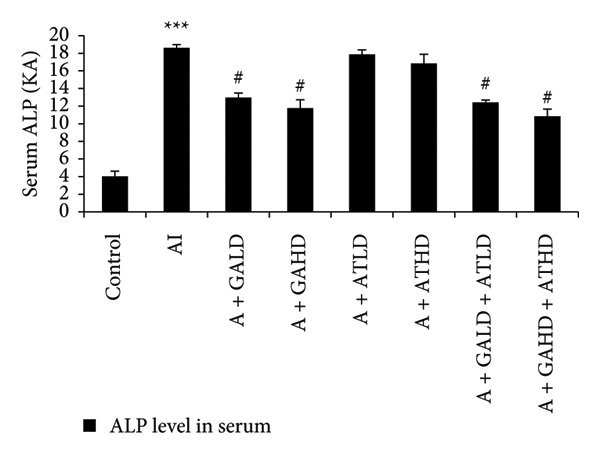
(c)

### 3.5. Assessment of Oxidative Stress Biomarkers

Evaluation of oxidative stress parameters provided insights into the antioxidant status of experimental animals. The adenine‐induced group showed a clear decline in the activities of SOD, CAT, and GSH compared to the control group. This indicates a weakened antioxidant defense. Conversely, GST and MDA levels were significantly higher, showing increased oxidative damage. Treatment with GA at doses of 100 mg/kg and 400 mg/kg, along with its combination with AT at the same doses, significantly restored the antioxidant enzymes SOD, CAT, and GSH to near‐normal values. This treatment also lowered the elevated GST and MDA levels. These findings suggest an overall improvement in oxidative balance after treatment (Figures 6(a), 6(b), 6(c), 7(a), and 7(b)).

Figure 6Graphs showing the (a) SOD, (b) CAT, and (c) GSH activities in kidney tissue of the rats of all the experimental groups: control (C), adenine‐induced (AI), adenine + gallic acid low dose (A + GALD), adenine + gallic acid high dose (A + GAHD), adenine + alpha‐tocopherol low dose (A + ATLD), adenine + alpha‐tocopherol high dose (A + ATHD), adenine + gallic acid low dose + alpha‐tocopherol low dose (A + GALD + ATLD), and adenine + gallic acid high dose + alpha‐tocopherol high dose (A + GAHD + ATHD). Animals (*n* = 6 per group) were given AI (320 mg/kg p.o.) to induce chronic kidney disease (CKD) and GALD (100 mg/kg p.o.), GAHD (400 mg/kg p.o.), ATLD (100 mg/kg p.o.), ATHD (400 mg/kg p.o.), GALD + ATLD (100 mg/kg p.o.), and GAHD + ATHD (400 mg/kg p.o.) orally as treatment. Kidney tissues were used to evaluate the SOD, CAT, and GST activities. Levels of SOD, CAT, and GST activities in the kidney are expressed as mean ± SEM (*n* = 6). The values are statistically significant at ^∗^ *p* < 0.05, ^∗∗^ *p* < 0.01, and ^∗∗∗^ *p* < 0.001 vs. the control group and ^#^ *p* < 0.05, ^##^ *p* < 0.01, and ^###^ *p* < 0.001 vs. the AI group, determined by one‐way ANOVA.

**Figure:**
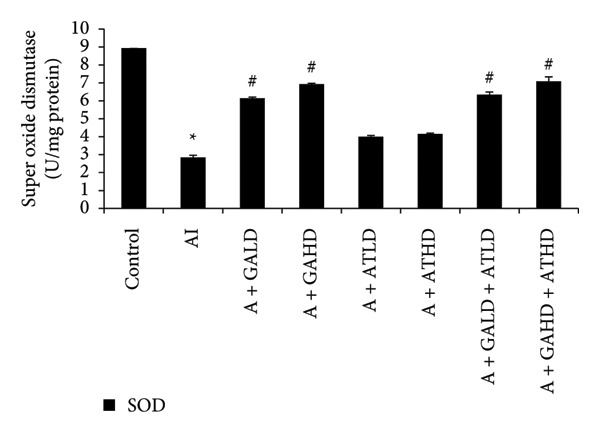
(a)

**Figure:**
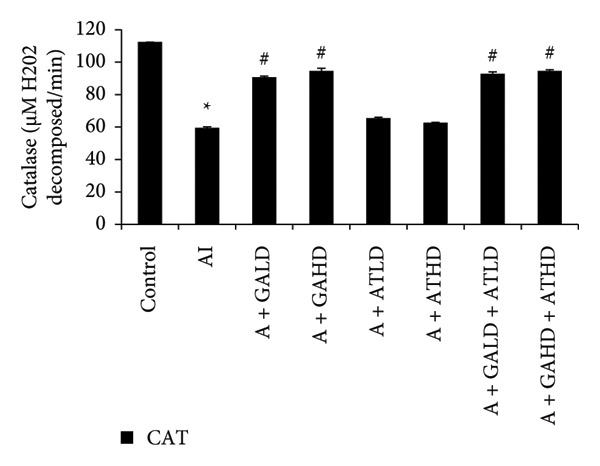
(b)

**Figure:**
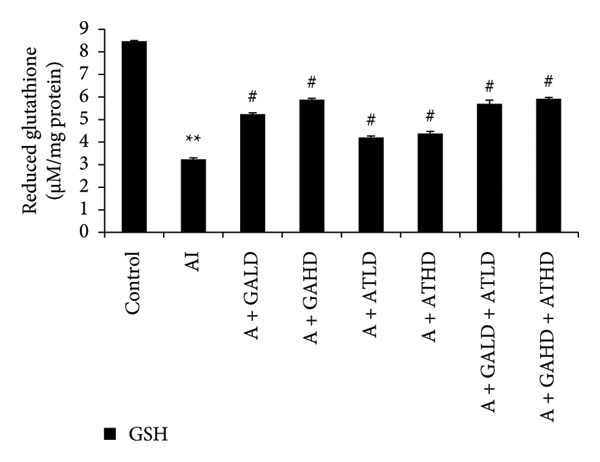
(c)

Figure 7Graphs showing the (a) MDA and (b) GST activities in kidney tissue of the rats of all the experimental groups: control (c), adenine‐induced (AI), adenine + gallic acid low dose (A + GALD), adenine + gallic acid high dose (A + GAHD), adenine + alpha‐tocopherol low dose (A + ATLD), adenine + alpha‐tocopherol high dose (A + ATHD), adenine + gallic acid low dose + alpha‐tocopherol low dose (A + GALD + ATLD), and adenine + gallic acid high dose + alpha‐tocopherol high dose (A + GAHD + ATHD). Animals (*n* = 6 per group) were given AI (320 mg/kg p.o.) to induce chronic kidney disease (CKD) and GALD (100 mg/kg p.o.), GAHD (400 mg/kg p.o.), ATLD (100 mg/kg p.o.), ATHD (400 mg/kg p.o.), GALD + ATLD (100 mg/kg p.o.), and GAHD + ATHD (400 mg/kg p.o.) orally as treatment. Kidney tissues were used to evaluate the MDA and GSH activities. Levels of MDA and GSH activities in the kidney are expressed as mean ± SEM (*n* = 6). The values are statistically significant at  ^∗^ *p* < 0.05,  ^∗∗^ *p* < 0.01, and  ^∗∗∗^ *p* < 0.001 vs. the control group and ^#^ *p* < 0.05, ^##^ *p* < 0.01, and ^###^ *p* < 0.001 vs. the AI group, determined by one‐way ANOVA.

**Figure:**
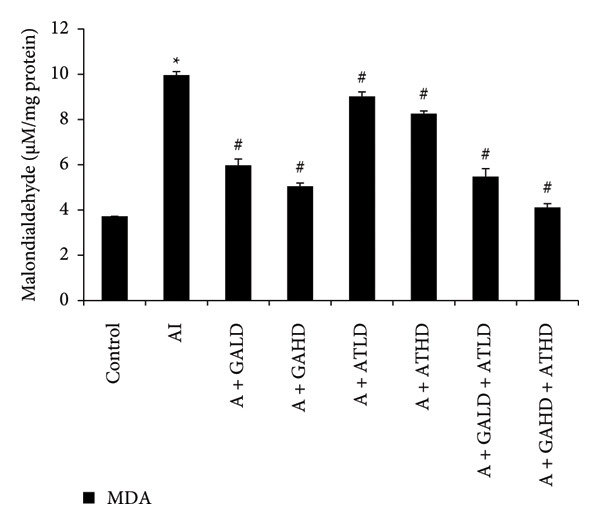
(a)

**Figure:**
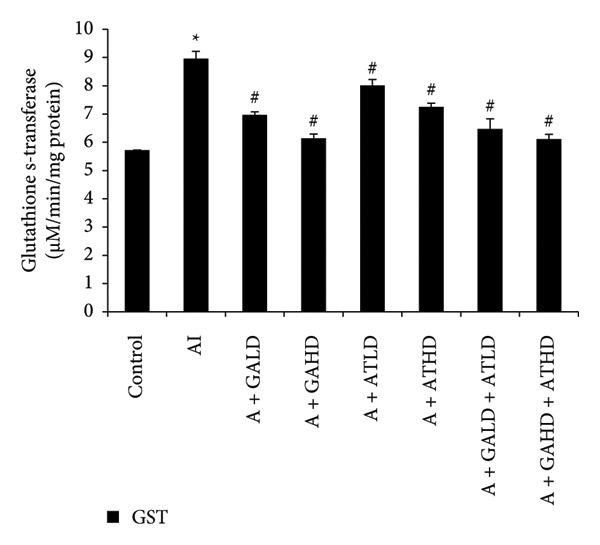
(b)

### 3.6. Histopathology

**Figure 8:**
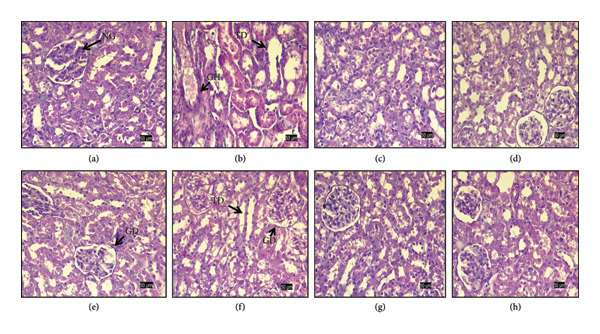
(a–h) Effects of gallic acid, alpha‐tocopherol, and their combined treatment on adenine‐induced CKD histopathological alteration in kidney. Histological sections of kidney tissue of the rats of all the experimental groups: (a) control (C), (b) adenine‐induced (AI), (c) adenine + gallic acid low dose (A + GALD), (d) adenine + gallic acid high dose (A + GAHD), (e) adenine + alpha‐tocopherol low dose (A + ATLD), (f) adenine + alpha‐tocopherol high dose (A + ATHD), (g) adenine + gallic acid low dose + alpha‐tocopherol low dose (A + GALD + ATLD), and (h) adenine + gallic acid high dose + alpha‐tocopherol high dose (A + GAHD + ATHD). (a) Normal control showing intact renal architecture and normal glomeruli (NG). (b) Group exhibiting severe tubular degeneration (TD), glomerular hypertrophy (GH), tubular dilation, and distorted renal architecture. (c and d) Groups showing reduced tubular damage and partial restoration of renal structure. (e and f) Distorted renal architecture and glomerular distortion (GD). (g and h) Groups demonstrating near‐normal glomeruli and tubules, indicating significant renoprotective effects.

Histological examination of H&E–stained kidney sections revealed clear structural variations among the experimental groups (Figure 8). The normal control group showed intact renal architecture with well‐defined glomeruli, normal Bowman’s space, and regularly arranged renal tubules, without any noticeable pathological alterations (Figure 8(a)). In contrast, the adenine‐treated groups (Figure 8(b)) exhibited pronounced renal damage, characterized by severe tubular degeneration and dilation, disruption of tubular epithelial lining, and enlarged, distorted glomeruli, leading to marked architectural disorganization.

Kidneys treated with doses of GA (Figures 8(c) and 8(d)) displayed appreciable histological improvement, with reduced tubular damage, partial restoration of epithelial integrity, and relatively preserved glomerular structure, though mild abnormalities were still evident, whereas AT‐treated groups (Figures 8(e) and 8(f)) could not restore the renal damage. The combined GA–AT‐treated groups (Figures 8(g) and 8(h)) showed near‐normal renal morphology, with well‐preserved glomeruli and tubules and minimal residual degenerative changes compared to the adenine‐treated group. Overall, adenine administration induced significant renal structural injury, whereas treatment with GA and combined GA–AT groups markedly attenuated these histopathological alterations and improved renal tissue organization.

### 3.7. Assessment of mRNA Levels of Kidney‐Related Markers

Quantitative PCR analysis evaluated the mRNA expression of key kidney marker genes, including Tnf, Tlr4, Igfbp7, Kl, Clu, Timp1, Vcam1, Timp2, Apoe, and Cystatin C, in the control, adenine‐induced CKD, and treatment groups. The adenine‐induced group showed a significant increase in the expression of all these genes compared to the control. This increase reflects the inflammatory and fibrotic responses linked to kidney injury. Treatment with GA, AT, and their combinations at doses of 100 mg/kg and 400 mg/kg taken orally significantly reduced the expression of these genes compared to the adenine group. Among the treatment groups, the GA and the combination of GA–AT were more effective in normalizing gene expression patterns. The observed decrease in transcript levels suggests that these compounds may help protect against adenine‐induced kidney dysfunction (Figures 9(a), 9(b), 9(c), 9(d), 10(a), 10(b), 10(c), 10(d), 11(a), and 11(b)).

Figure 9Relative mRNA expression levels of (a) Tnf, (b) Tlr4, (c) Igfbp7, and (d) Kl in kidneys of rats of all the experimental groups: control (C), adenine‐induced (AI), adenine + gallic acid low dose (A + GALD), adenine + gallic acid high dose (A + GAHD), adenine + alpha‐tocopherol low dose (A + ATLD), adenine + alpha‐tocopherol high dose (A + ATHD), adenine + gallic acid low dose + alpha‐tocopherol low dose (A + GALD + ATLD), and adenine + gallic acid high dose + alpha‐tocopherol high dose (A + GAHD + ATHD). Animals (*n* = 6 per group) were given AI (320 mg/kg p.o.) to induce chronic kidney disease (CKD) and GALD (100 mg/kg p.o.), GAHD (400 mg/kg p.o.), ATLD (100 mg/kg p.o.), ATHD (400 mg/kg p.o.), GALD + ATLD (100 mg/kg p.o.), and GAHD + ATHD (400 mg/kg p.o.) orally as treatment. Levels of transcripts in the experimental groups are expressed as fold changes relative to the control group after being normalized against the GAPDH standard. Data shown as mean ± SE (*n* = 6) of relative concentrations (2^−ΔΔCt^ method; Livak et al., 2001). The values are statistically significant at ^∗^ *p* < 0.05, ^∗∗^ *p* < 0.01, and ^∗∗∗^ *p* < 0.001 as compared to the control group and ^#^ *p* < 0.05, ^##^ *p* < 0.01, and ^###^ *p* < 0.001 as compared to the AI group (one‐way ANOVA).

**Figure:**
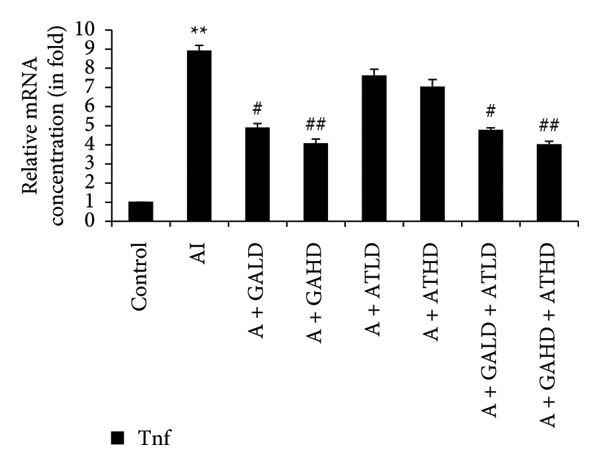
(a)

**Figure:**
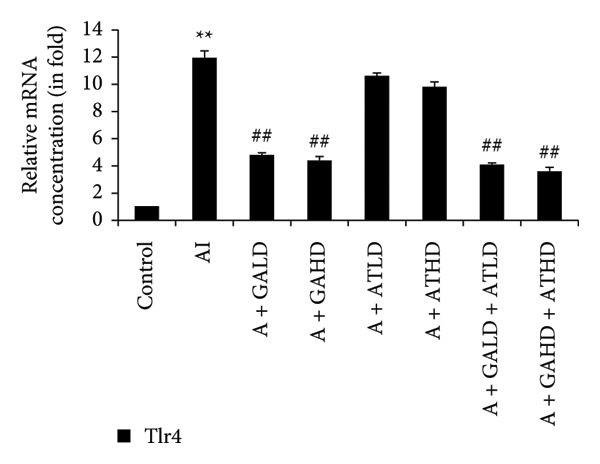
(b)

**Figure:**
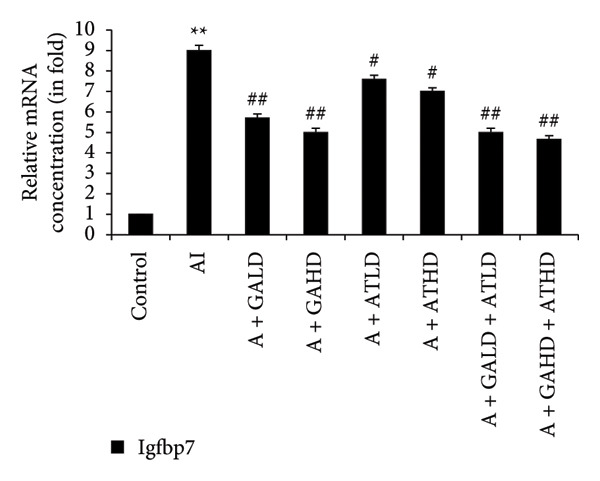
(c)

**Figure:**
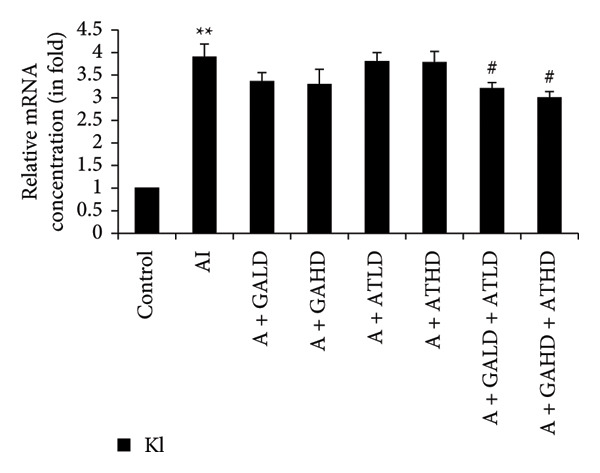
(d)

Figure 10Relative mRNA expression levels of (a) Clu, (b) Timp1, (c) Vcam1, and (d) Timp2 in kidneys of rats of all the experimental groups: control (C), adenine‐induced (AI), adenine + gallic acid low dose (A + GALD), adenine + gallic acid high dose (A + GAHD), adenine + alpha‐tocopherol low dose (A + ATLD), adenine + alpha‐tocopherol high dose (A + ATHD), adenine + gallic acid low dose + alpha‐tocopherol low dose (A + GALD + ATLD), and adenine + gallic acid high dose + alpha‐tocopherol high dose (A + GAHD + ATHD). Animals (*n* = 6 per group) were given AI (320 mg/kg p.o.) to induce chronic kidney disease (CKD) and GALD (100 mg/kg p.o.), GAHD (400 mg/kg p.o.), ATLD (100 mg/kg p.o.), ATHD (400 mg/kg p.o.), GALD + ATLD (100 mg/kg p.o.), and GAHD + ATHD (400 mg/kg p.o.) orally as treatment. Levels of transcripts in the experimental groups are expressed as fold changes relative to the control group after being normalized against the GAPDH standard. Data shown as mean ± SE (*n* = 6) of relative concentrations (2^−ΔΔCt^ method; Livak et al., 2001). The values are statistically significant at ^∗^ *p* < 0.05, ^∗∗^ *p* < 0.01, and ^∗∗∗^ *p* < 0.001 as compared to the control group and ^#^ *p* < 0.05, ^##^ *p* < 0.01, and ^###^ *p* < 0.001 as compared to the AI group (one‐way ANOVA).

**Figure:**
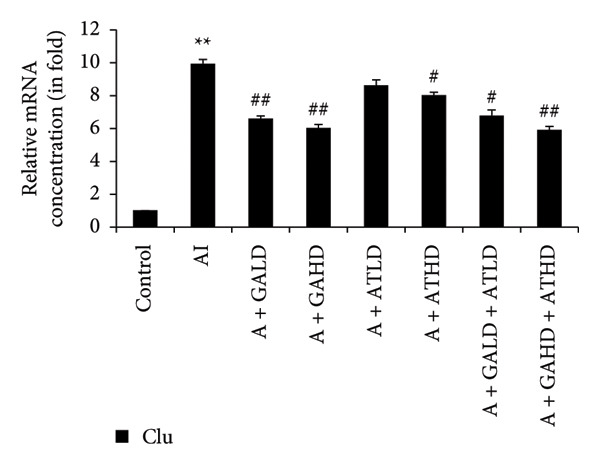
(a)

**Figure:**
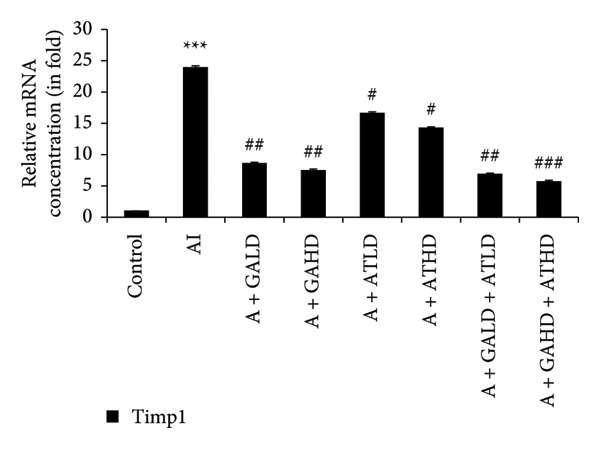
(b)

**Figure:**
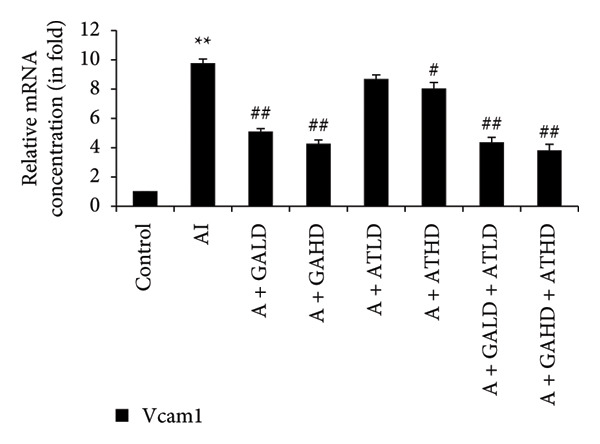
(c)

**Figure:**
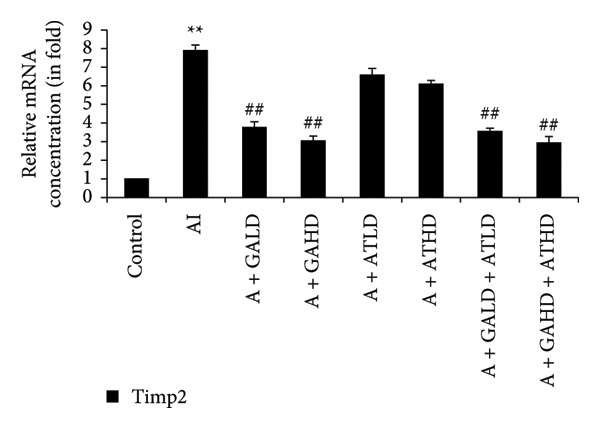
(d)

Figure 11Relative mRNA expression levels of (a) Apoe and (b) Cystatin C in kidneys of rats of all the experimental groups: control (C), adenine‐induced (AI), adenine + gallic acid low dose (A + GALD), adenine + gallic acid high dose (A + GAHD), adenine + alpha‐tocopherol low dose (A + ATLD), adenine + alpha‐tocopherol high dose (A + ATHD), adenine + gallic acid low dose + alpha‐tocopherol low dose (A + GALD + ATLD), and adenine + gallic acid high dose + alpha‐tocopherol high dose (A + GAHD + ATHD). Animals (*n* = 6 per group) were given AI (320 mg/kg p.o.) to induce chronic kidney disease (CKD) and GALD (100 mg/kg p.o.), GAHD (400 mg/kg p.o.), ATLD (100 mg/kg p.o.), ATHD (400 mg/kg p.o.), GALD + ATLD (100 mg/kg p.o.), and GAHD + ATHD (400 mg/kg p.o.) orally as treatment. Levels of transcripts in the experimental groups are expressed as fold changes relative to the control group after being normalized against the GAPDH standard. Data shown as mean ± SE (*n* = 6) of relative concentrations (2^−ΔΔCt^ method; Livak et al., 2001). The values are statistically significant at ^∗^ *p* < 0.05, ^∗∗^ *p* < 0.01, and ^∗∗∗^ *p* < 0.001 as compared to the control group and ^#^ *p* < 0.05, ^##^ *p* < 0.01, and ^###^ *p* < 0.001 as compared to the AI group (one‐way ANOVA).

**Figure:**
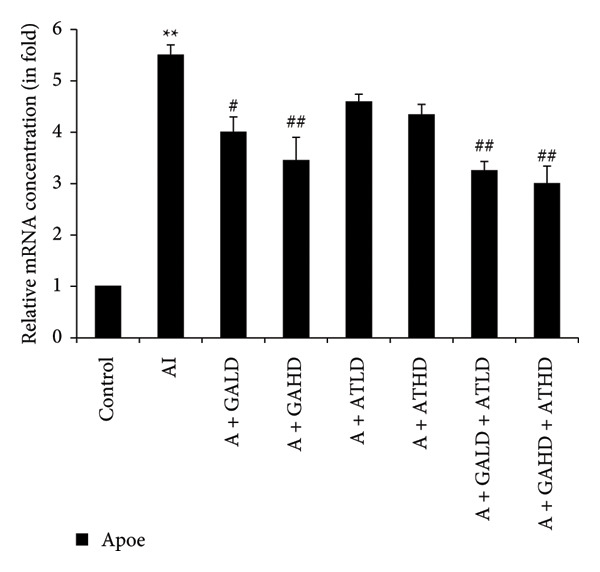
(a)

**Figure:**
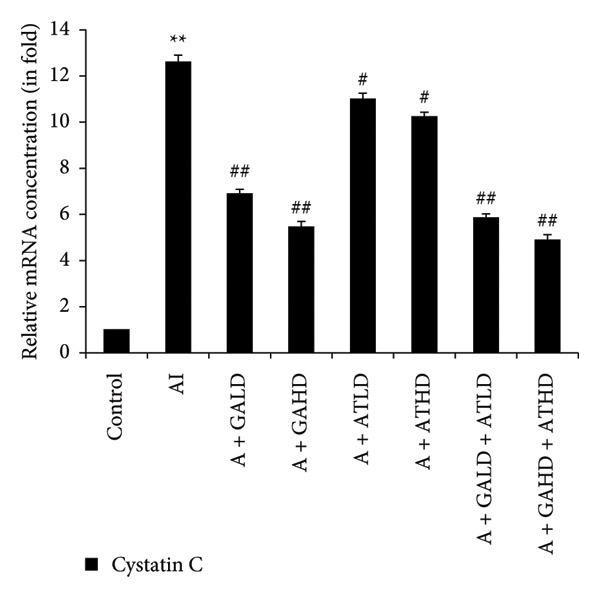
(b)

### 3.8. Molinspiration

The Molinspiration analysis was performed to evaluate the molecular and drug‐like properties of GA and AT. The results showed that both compounds have favorable molecular features that support good oral bioavailability. Their physicochemical behavior matched general drug‐like standards, displaying only minor deviations. This suggests they could be efficiently absorbed and distributed in biological systems (Table 3).

**Table 3:** Molecular properties of the ligands evaluated using Molinspiration.

Ligands	ChemSpider ID	miLogP	H‐bond acceptor	H‐bond donor	Mol wt. (g/mol)	No. of violation	TPSA
Gallic acid	361	0.59	5	4	170.12	0	97.98
Alpha‐tocopherol	14,265	9.04	2	1	430.72	1	29.46

### 3.9. In Silico Docking Evaluation

In silico docking experiments were performed to analyze the interaction patterns of compounds (GA and AT) with target proteins (Tnf, Tlr4, Igfbp7, Kl, Clu, Timp1, Vcam1, Timp2, Apoe, and Cystatin C) of our study. The analysis of protein–ligand complexes focused on parameters such as minimum binding free energy (ΔG), hydrogen bond count, and hydrophobic interactions. The present study confirmed that selected compounds, GA (Figures 12, 13, and 14) and AT (Figures 15, 16, and 17), interacted and displayed promising binding affinities toward all 10 target proteins (Tables S4 and S5). It was observed that among all 10 proteins, the results revealed that GA possessed the most effective binding affinity toward Timp2 through hydrogen‐bonding capability with ARG111:HH21, PHE124:HN, SER38:OG, CYS122:O, ALA109:O, and hydrophobic interactions with its ALA34 residue, yielding a binding energy (ΔG) of −4.97 kcal/mol, whereas AT showed a strong binding affinity to Kl, marked by hydrogen bonding with ASN514:OD1 and hydrophobic interactions with the residues TRP852, LYS501, PRO742, LEU502, TYR434, PHE498, and PHE508, yielding a binding free energy (ΔG) of −8.14 kcal/mol.

Figure 12Best docking conformations of gallic acid with target proteins (a) Timp1, (b) Igfbp7, (c) Kl, and (d) Apoe, obtained using AutoDock. Active site residues are depicted as lines and labeled with their respective amino acid positions.

**Figure:**
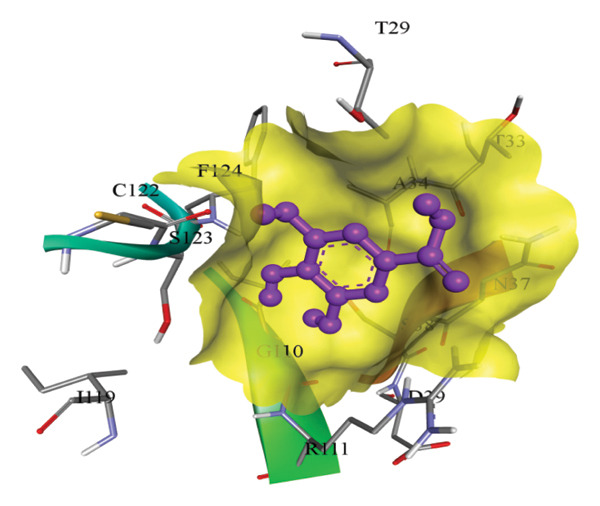
(a)

**Figure:**
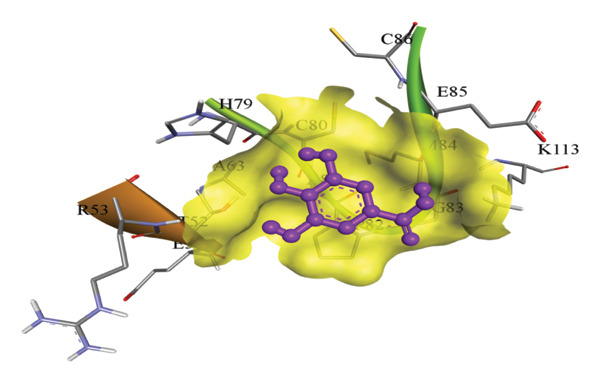
(b)

**Figure:**
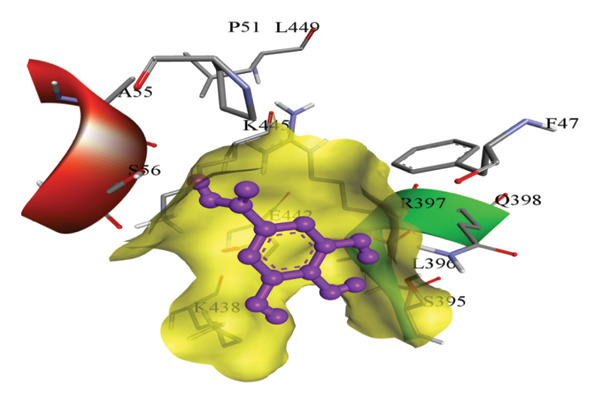
(c)

**Figure:**
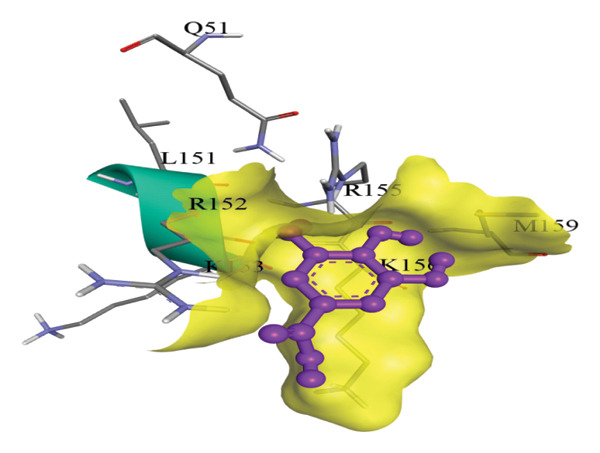
(d)

Figure 13Best docking conformations of gallic acid with target proteins (a) Timp2, (b) Vcam1, (c) Cystatin C, and (d) Tnf, obtained using AutoDock. Active site residues are depicted as lines and labeled with their respective amino acid positions.

**Figure:**
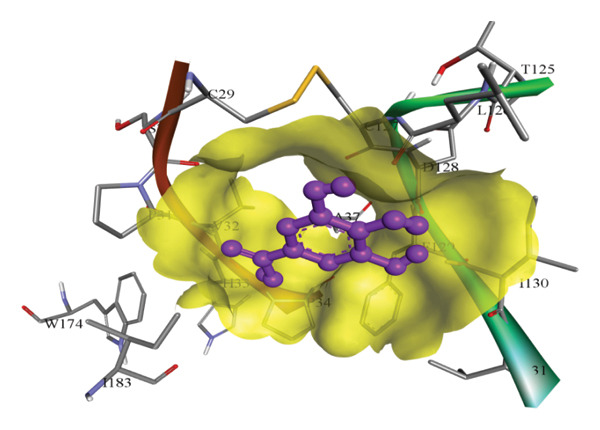
(a)

**Figure:**
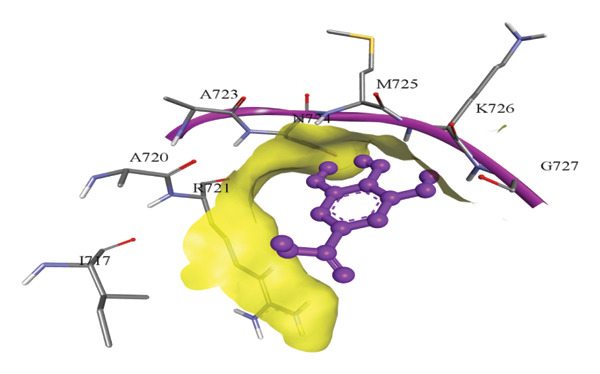
(b)

**Figure:**
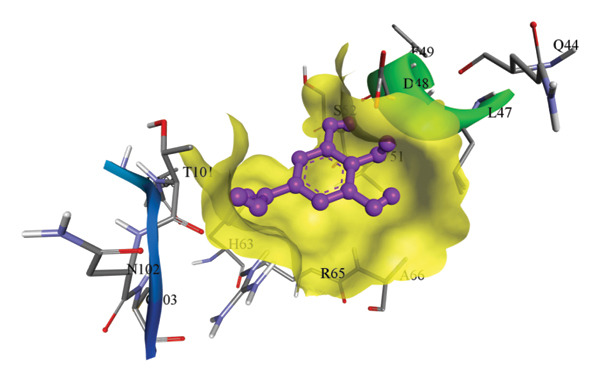
(c)

**Figure:**
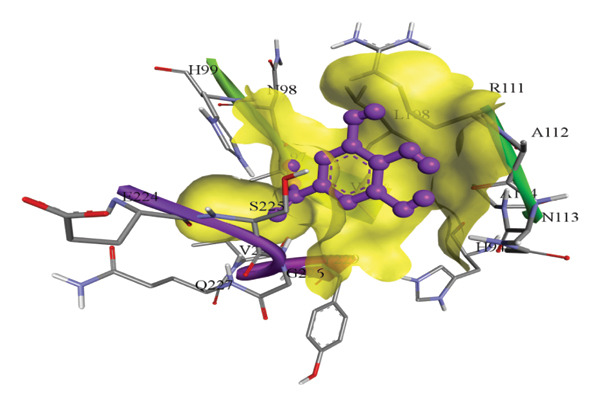
(d)

Figure 14Best docking conformations of gallic acid with target proteins (a) Tlr4 and (b) Clu, obtained using AutoDock. Active site residues are depicted as lines and labeled with their respective amino acid positions.

**Figure:**
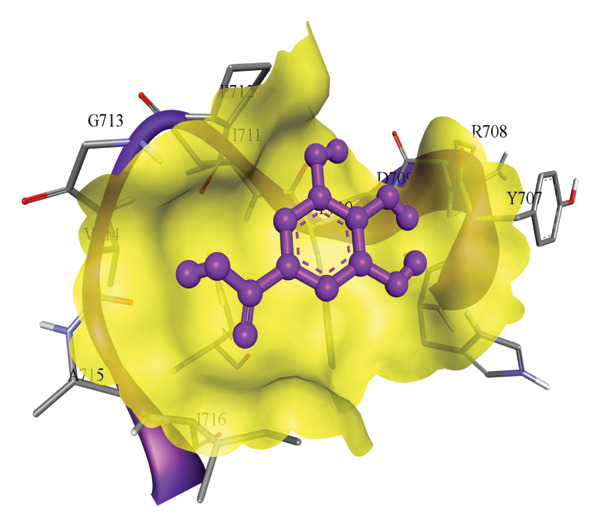
(a)

**Figure:**
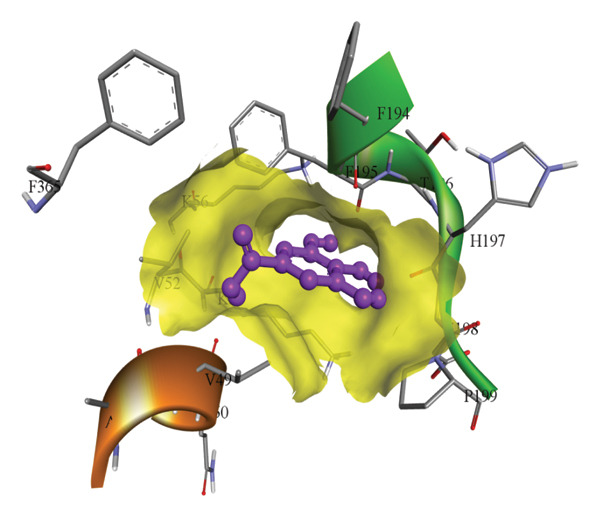
(b)

Figure 15Best docking conformations of alpha‐tocopherol with target proteins (a) Kl, (b) Timp2, (c) Clu, and (d) Igfbp7, obtained using AutoDock. Active site residues are depicted as lines and labeled with their respective amino acid positions.

**Figure:**
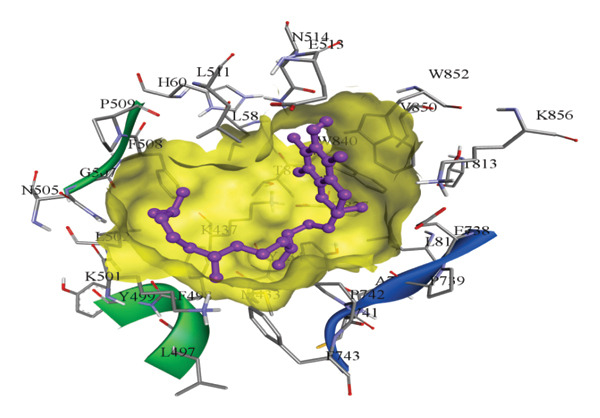
(a)

**Figure:**
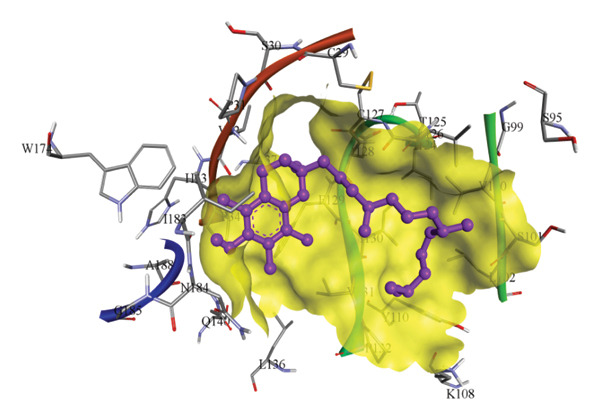
(b)

**Figure:**
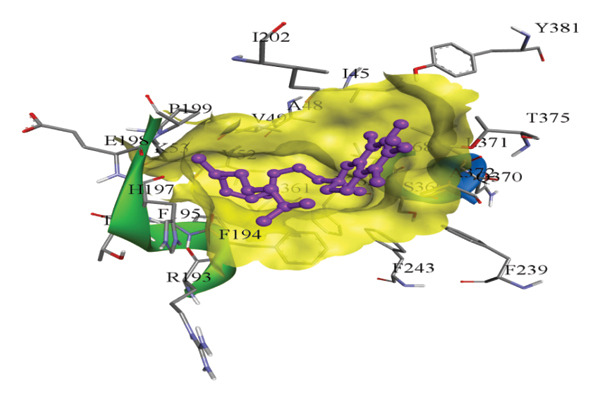
(c)

**Figure:**
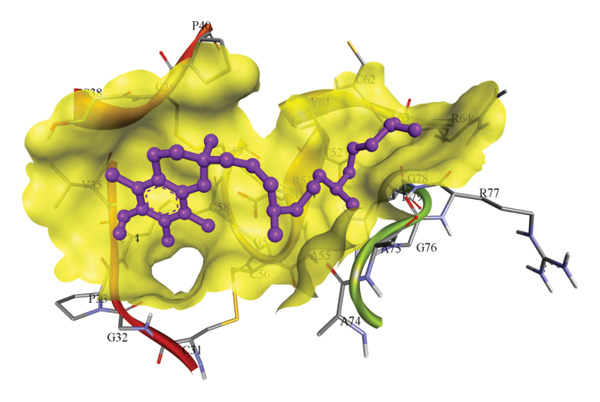
(d)

Figure 16Best docking conformations of alpha‐tocopherol with target proteins (a) Tlr4, (b) Vcam1, (c) Timp1, and (d) Cystatin C, obtained using AutoDock. Active site residues are depicted as lines and labeled with their respective amino acid positions.

**Figure:**
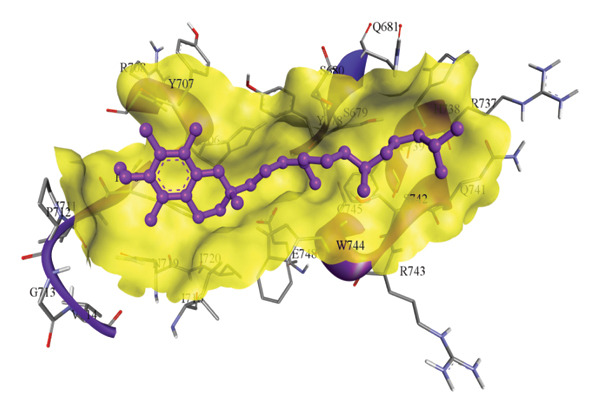
(a)

**Figure:**
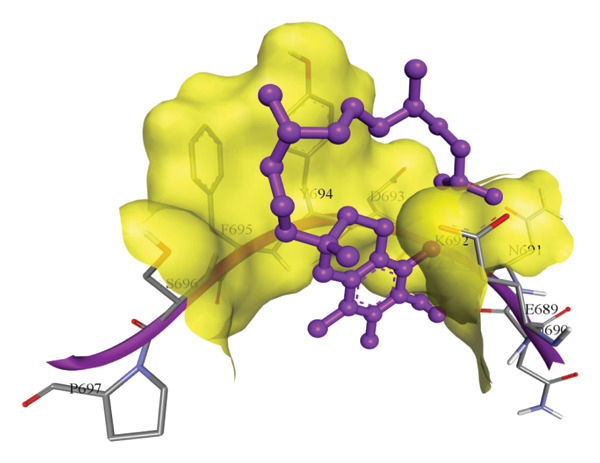
(b)

**Figure:**
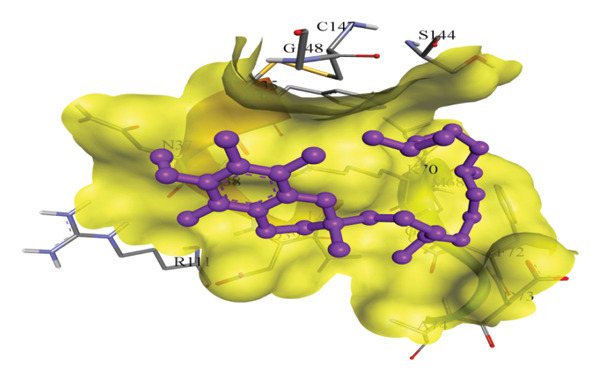
(c)

**Figure:**
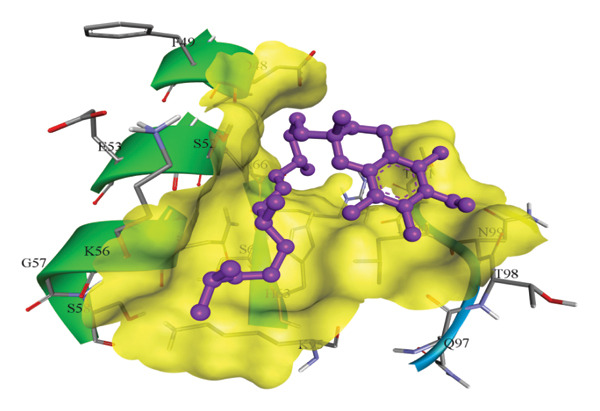
(d)

Figure 17Best docking conformations of alpha‐tocopherol with target proteins (a) Tnf and (b) Apoe, obtained using AutoDock. Active site residues are depicted as lines and labeled with their respective amino acid positions.

**Figure:**
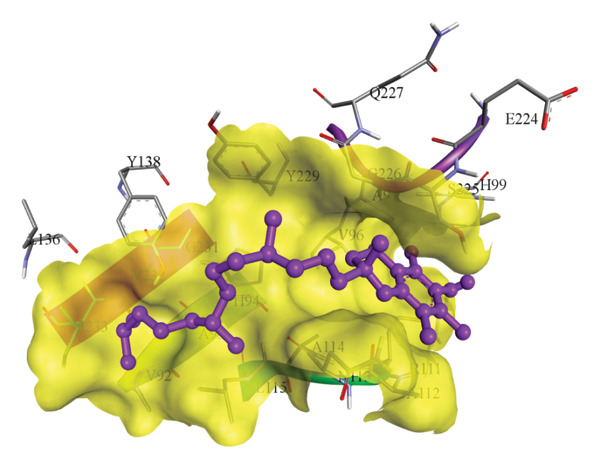
(a)

**Figure:**
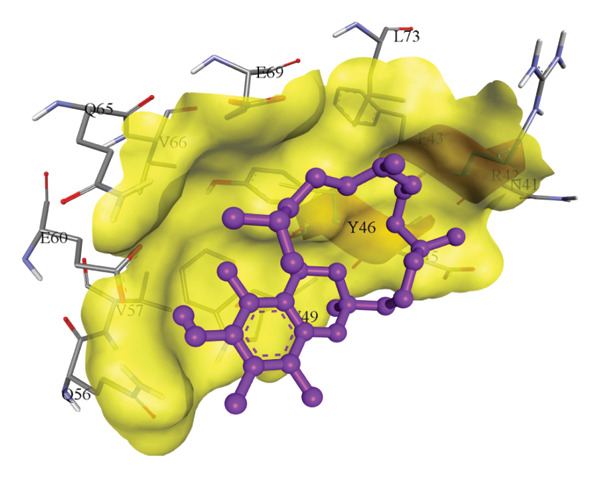
(b)

## 4. Discussion

In the present study, CKD was experimentally induced, leading to a gradual decline in renal function accompanied by biochemical and histological alterations. CKD is known to involve complex pathological processes, in which oxidative stress and inflammatory responses play major contributory roles. The observations made in this work align with this understanding, as the adenine‐challenged group showed clear evidence of renal impairment and heightened oxidative burden. Animal models induced with adenine provide valuable insights into CKD pathophysiology and potential therapeutic strategies. Through this assessment, we sought to determine the ameliorative capability of the selected natural compounds, GA, AT, and their combined doses in a male Wistar rat model of adenine‐induced CKD.

The CKD condition in rats was produced using adenine, which is widely used to reproduce the oxidative and inflammatory disturbance characteristics of human CKD. In our study, the animals received a diet containing 0.75% adenine for a period of 4 weeks. This regimen is known to gradually impair renal function and is commonly employed to investigate potential kidney‐protective interventions [40, 41]. Subsequently, we evaluated the effects of GA, AT, and their combination on mitigating these changes. Throughout the experimental period, the rats receiving adenine showed a marked reduction in body weight, reflecting the systemic stress associated with CKD. However, administration of GA and AT helped in recovering the lost weight, indicating an improvement in overall health status. The relative kidney weight, which was elevated in the adenine group due to renal swelling and structural damage, was also brought closer to normal values following the treatments, suggesting protective effects on kidney morphology and function. Additionally, adenine administration resulted in excessive water intake and increased urine volume, a common feature of renal impairment. These alterations were gradually corrected in the treatment groups, demonstrating a restoration of fluid balance and renal concentrating ability [23, 42]. We have also analyzed the urine markers urea, creatinine, uric acid, and albumin of all the experimental groups, and it was observed that GA and combined doses of GA–AT could outperform the single doses of AT and mitigate the effects of the adenine diet. Urea, uric acid, creatinine, and albumin are commonly evaluated biochemical indicators of renal status. Elevated levels of urea and creatinine generally reflect impaired filtration efficiency, whereas changes in uric acid levels indicate alterations in metabolic handling and renal excretion. The presence of albumin in urine is a sign of compromised glomerular integrity. Assessing these parameters allows us to identify the extent of kidney dysfunction and determine the therapeutic effectiveness of interventions [43–46]. Clinically, biochemical markers such as BUN, serum creatinine, urea, uric acid, albumin, phosphorus, and NO provide important information regarding kidney function, particularly glomerular filtration efficiency and tubular reabsorption capacity. These parameters are routinely applied in the assessment and staging of chronic renal impairment. In general, elevated BUN and urea levels suggest reduced filtration efficiency and possible fluid imbalance. Serum creatinine, produced from muscle metabolism, remains one of the most reliable indicators of the glomerular filtration rate and overall renal functional status. Uric acid levels are indicative of purine metabolism and can highlight conditions such as gout. Albumin is generally retained within the bloodstream, and its appearance in urine is therefore indicative of compromised glomerular integrity. Disturbances in phosphorus levels, which are normally regulated by renal excretion, are commonly associated with altered mineral balance and bone complications in kidney disorders. Nitrogenous waste, reflected in BUN measurements, provides insight into protein turnover and renal clearance efficiency. The evaluation of these biochemical markers is essential for identifying the extent of kidney impairment and for informing appropriate clinical management [47–50].

Adenine intake resulted in significant renal dysfunction, as shown by raised levels of creatinine, urea, uric acid, phosphorus, NO, and BUN, together with a reduction in circulating albumin. These alterations point to impaired kidney filtration and protein loss [51]. Following treatment with GA and AT, these abnormalities were substantially improved. The previously elevated markers declined, and albumin concentrations increased, suggesting recovery of renal function. Overall, the biochemical data indicate that GA and AT contributed to restoring kidney performance and maintaining glomerular integrity in adenine‐induced CKD [52]. In our study, oxidative stress was assessed by examining the activity of key antioxidant enzymes (SOD, CAT, and GST, along with GSH levels) and MDA as an indicator of lipid peroxidation. Rats in the adenine group showed a clear reduction in SOD, CAT, and GSH, reflecting a compromised antioxidant defense system. At the same time, GST and MDA levels were elevated, suggesting increased oxidative load and cellular membrane damage within the kidney. Administration of GA, either alone or together with AT, helped to counter these disturbances. The treatments enhanced SOD and CAT activity and restored GSH levels, whereas GST and MDA values declined toward those seen in healthy controls. This pattern indicates a reduction in oxidative stress and a strengthening of the kidney’s intrinsic antioxidant capacity. These findings suggest that GA and the GA–AT combination play a protective role in the CKD condition by re‐establishing redox balance and reducing oxidative injury, which is an important factor in preventing further renal deterioration [53, 54].

Our study underscores the therapeutic potential of GA and AT, alone and in combination, in attenuating oxidative damage and preserving kidney function in the CKD mediated by adenine models. The present findings suggest that these compounds hold promise as therapeutic candidates for the management of CKD. However, additional research is necessary to clarify their molecular mechanisms and to determine their relevance in clinical contexts. GA and AT are distinguished by their strong antioxidant capacities. In particular, GA neutralizes ROS, thereby lowering oxidative stress and protecting cellular structures from associated damage. AT, a form of vitamin E, interrupts chain reactions of lipid peroxidation by donating hydrogen atoms to free radicals, thereby reducing oxidative damage in biological membranes. These antioxidant actions of GA and AT contribute to their potential therapeutic benefits in mitigating oxidative stress‐related diseases. Studies underscore their efficacy in experimental models, highlighting their roles in cellular protection and potential applications in oxidative stress‐related conditions [55, 56]. Histopathological evaluation of kidney sections provided important insight into the extent of renal injury due to adenine‐induced CKD and the protective effects of the treatments employed in this study. The normal control group exhibited well‐maintained renal architecture, reflecting normal glomerular and tubular integrity, which served as a baseline for comparison with the experimental groups. Administration of adenine resulted in marked renal structural alterations, including severe tubular degeneration, tubular dilation, and glomerular hypertrophy. These changes are characteristic of chronic renal injury and indicate substantial impairment of renal tissue organization. Disruption of tubular epithelial integrity and distortion of glomerular structure observed in the adenine‐treated groups suggest compromised filtration and tubular function, consistent with progressive kidney damage. In contrast, treatment with GA led to noticeable histological improvement. Reduced tubular degeneration and partial restoration of epithelial lining indicate attenuation of renal injury, although the persistence of mild abnormalities suggests incomplete recovery. These findings highlight the ability of the standard treatment to limit, but not entirely prevent, adenine‐induced renal damage. Notably, kidneys from the combination groups of GA–AT showed near‐normal histological features, with substantial preservation of glomerular and tubular architecture. The marked reduction in tubular and glomerular alterations compared to the adenine‐treated group suggests a pronounced renoprotective effect of the treatment [40, 57]. Overall, these histopathological observations support the conclusion that adenine induces significant renal injury, whereas therapeutic intervention effectively mitigates structural damage and promotes restoration of renal tissue organization.

The qPCR study in our research dealt with the important key kidney markers: Tnf, Tlr4, Igfbp7, Kl, Clu, Timp1, Vcam1, Timp2, Apoe, and Cystatin C. These genes (tumor necrosis factor [Tnf], Toll‐like receptor 4 [Tlr4], tissue inhibitor of metalloproteinases 1 [Timp1], insulin‐like growth factor binding protein 7 [Igfbp7], Klotho [Kl], Clusterin C, Timp2, vascular cell adhesion molecule 1 [Vcam1], apolipoprotein E [Apoe], and Cystatin C) play critical roles in kidney health by influencing inflammation, immune response, fibrosis, and overall renal function. TNF and TLR4 are involved in regulating immune responses during kidney injury [57, 58], whereas IGFBP7 serves as a marker for acute kidney injury [59, 60]. Kl and Clu contribute antiaging and antifibrotic effects in kidneys [61, 62] and Timp1 and Timp2 participate in remodeling the extracellular matrix, impacting renal fibrosis [63, 64]. Vcam1 facilitates leukocyte recruitment in renal diseases [65, 66], Apoe influences lipid metabolism and renal function risk [60, 67], and Cystatin C acts as a biomarker for renal function, indicating impaired kidney function when elevated [53, 68, 69]. Understanding these genes provides insights into the complex molecular pathways affecting kidney health and offers potential avenues for therapeutic intervention in renal diseases. We have observed that the group in which CKD was induced by adenine displayed noticeably higher levels of expression of the relative mRNA of all the genes, but with treatment of GA, AT, and GA–AT, we noticed that the increased mRNA expression of the genes was significantly lowered back in comparison with the group receiving adenine. However, among the three doses, GA and combined doses of GA–AT did better performance than that of AT. These findings showed the potential of GA and AT in mitigating the effects of adenine‐induced CKD in Wistar rats.

The molecular properties and bioactivity scores of GA and AT were evaluated using the Molinspiration software, adhering to the five rules of Lipinski [36]. Both compounds demonstrated promising scores, highlighting their viability as pharmacologically active candidates. Subsequently, molecular docking evaluations were conducted to investigate their binding interactions with key kidney biomarkers: Tnf, Tlr4, Igfbp7, Kl, Clu, Timp1, Vcam1, Timp2, Apoe, and Cystatin C. This analysis aims to elucidate how GA and AT could potentially modulate these biomarkers, providing insights into their therapeutic mechanisms for kidney‐related conditions. In our findings, we have found that GA and AT both interacted with all 10 genes with good binding energies. Docking studies predict how these compounds bind to the active sites of these markers, influencing their activity and potentially modulating pathways involved in kidney inflammation, fibrosis, and dysfunction.

## 5. Conclusion

In conclusion, our study demonstrates that GA, alone and in combination with AT, effectively ameliorates CKD mediated by adenine in male Wistar rats. GA, recognized for its potent antioxidant activity, played a key role in restoring renal function and reducing oxidative damage. The observed improvement in kidney physiology, along with diminished oxidative stress, highlights its protective influence on renal tissues and its ability to preserve cellular integrity. The combined treatment of GA and AT further exhibited synergistic effects, enhancing these outcomes and modulating critical molecular markers associated with kidney function. Collectively, these natural compounds demonstrate promise in restoring renal performance, attenuating oxidative stress, and regulating molecular pathways linked to CKD progression. Therefore, our findings provide a strong foundation for considering GA and AT as potential therapeutic candidates for the management of CKD, pending further validation through clinical and mechanistic studies.

**Nomenclature**
ADT: AutoDockTools
ALP: Alkaline phosphatase
ALT: Alanine aminotransferase
Apoe: Apolipoprotein E ARRIVE Animal Research: Reporting of In Vivo Experiments
AST: Aspartate aminotransferase
AT: Alpha‐tocopherol
BUN: Blood urea nitrogen
CAT: Catalase
CCSEA: Committee for Control and Supervision of Experiments on Animals
cDNA: Complementary deoxyribonucleic acid
CKD: Chronic kidney disease
Clu: Clusterin
DNA: Deoxyribonucleic acid
DPX: Dibutylphthalate polystyrene xylene
GA: Gallic acid
GAPDH: Glyceraldehyde 3‐phosphate dehydrogenase
GFR: Glomerular filtration rate
GSH: Reduced glutathione
GST: Glutathione S‐transferase
Igfbp7: Insulin‐like growth factor binding protein 7
IP: Intraperitoneal
Kl: Klotho
MDA: Malondialdehyde
mRNA: Messenger ribonucleic acid
ROS: Reactive oxygen species
NO: Nitric oxide
PCR: Polymerase chain reaction
qPCR: Quantitative polymerase chain reaction
RNA: Ribonucleic acid
SOD: Superoxide dismutase
Timp1: TIMP metallopeptidase inhibitor 1
Timp2: Tissue inhibitor of metalloproteinases 2
Tlr4: Toll‐like receptor 4
Tnf: Tumor necrosis factor
Vcam1: Vascular cell adhesion molecule 1

## Ethics Statement

The present study was performed in compliance with the Animal Research: Reporting of In Vivo Experiments (ARRIVE) guidelines and the regulations set forth by the Committee for the Control and Supervision of Experiments on Animals (CCSEA). All experimental procedures received approval from the Institutional Animal Ethics Committee of Gauhati University, Guwahati, India (Approval No. IAEC/Per/2019/PP‐IAEC/2022‐4).

## Disclosure

All authors have read and approved the final version of the manuscript.

## Conflicts of Interest

The authors declare no conflicts of interest.

## Author Contributions

Momita Rani Baro designed and conducted the experiments, analyzed and interpreted the data, and prepared the initial draft of the manuscript. Manas Das contributed to the conceptualization of the study and revised the manuscript by incorporating additional valuable content. Kishore Sarma performed the bioinformatic analyses and assisted in the interpretation of the findings. Leena Das, Aashis Dutta, Ananya Chetia, and Pliza Kalita reviewed, proofread, and contributed to enhancing the overall quality of the manuscript.

## Funding

The work has received no funding.

## Supporting Information

Table S1: Primer sequences used for real‐time quantitative PCR (qPCR) analysis.

Table S2: PubChem identification numbers of SDF files corresponding to the 3D conformers of the selected ligands.

Table S3: Protein identification details along with their active binding site information.

Table S4: Binding energy values of gallic acid with the selected target proteins.

Table S5: Binding energy values of alpha‐tocopherol with the selected target proteins.

## Supporting information


**Supporting Information** Additional supporting information can be found online in the Supporting Information section.

## Data Availability

The data supporting the findings of this study can be obtained from the corresponding author on request.
