# Supplementary material for: Therapeutic Effects of Gallic Acid and Alpha‐Tocopherol on Adenine‐Induced Chronic Kidney Disease in Male Wistar Rats
Source: Biochem Res Int. 2026 Jan 20;2026:9220531. doi: 10.1155/bri/9220531 (PMC12818050; doi:10.1155/bri/9220531)
Supplement: Supplementary file 1 — Supporting Information Additional supporting information can be found online in the Supporting Information section. [file BRI-2026-9220531-s001.docx]

**Supplementary file**

**Table S1.** Primer sequences used for real-time quantitative PCR (qPCR) analysis.

| ***Gene name*** | **Forward Primer sequence** | **Reverse Primer sequence** | **Accession number** |
| --- | --- | --- | --- |
| ***Tnf*** | ATGGGCTCCCTCTCATCAGT | GCTTGGTGGTTTGCTACGAC | NM_012675.3 |
| ***Tlr4*** | TCTGGGTTTCTGCTGTGGAC | TGCTACTTCCTTGTGCCCTG | NM_019178.2 |
| ***Igfbp7*** | GCGTCTAGCCACCCCATCAT | GGCATCGGAAGAGGAGGAAG | NM_001013048.1 |
| ***Kl*** | TCAAGGATGGTGGCGGTTTT | GGTGGGCATAGGTGCGTAAT | NM_031336.1 |
| ***Clu*** | AGCAATCGGGTGAAGTAAACAC | GGAGAATCTTCATGGTGTGGC | NM_053021.2 |
| ***Timp1*** | GCCTCTGGCATCCTCTTGTT | AGAAAGCTGTCTGTGGGTGG | U06179.1 |
| ***Vcam1*** | CCTCCTTGGTAATACCCGCC | TTGACTTCTGTGCCTCCACC | NM_012889.1 |
| ***Timp2*** | GTGGCCAATTGAAAAGCCTCTG | TAGCGTGAACCCACTTGGATGA | NM_021989.2 |
| ***Apoe*** | ATTGCTGACAGGTATGGGGC | ACAGCTGCTCAGGGCTATTG | J02582.1 |
| ***Cystatin C*** | CTCTATCTCTCACAATTGGGTA | CAGTAGCATCAAGGAGCGCAG | NM_012837.1 |
| ***Gapdh*** | AGTGCCAGCCTCGTCTCATA | GGTAACCAGGCGTCCGATAC | NM_017008.4 |

**Table S2.** PubChem identification numbers of SDF files corresponding to the 3D conformers of the selected ligands.

| **Ligands** | **PubChem ID** |
| --- | --- |
| Gallic acid | PubChem CID-370 |
| Alpha-Tocopherol | PubChem CID-14985 |

**Table S3.** Protein identification details along with their active binding site information.

| **Proteins** | **ID** | **Binding site residues** |
| --- | --- | --- |
| Tumour necrosis factor (Tnf) | UniProt ID P16599 | VAL92, ALA93, HIS94, ASN113, LEU115, LEU136, TYR138, TYR197, GLY199, GLY226, TYR229, ILE233 |
| Toll like receptor 4 (Tlr4) | UniProt ID Q9QX05 | TYR678, SER680, HIS706, PHE710, ILE716, SER742, TRP744, CYS745, GLU748 |
| Insulin like growth factor binding protein 7 (Igfbp7) | UniProt ID F1M9B2 | GLY71, GLY78, HIS79, CYS80, CYS86 |
| Klotho (Kl) | UniProt ID Q9Z2Y9 | LEU16, GLU54, GLY57, LEU58, HIS60, MET433, TYR434, LYS437, PHE498, LYS501, LEU502, GLY507, PHE508, PRO509, GLU513, ASN514, GLU738, PRO739, ALA740, PRO742, PHE743, LEU815, ASP837, THR839, TRP840, LEU841, VAL849, PRO851, TRP852, TYR891, ASN895, GLU899 |
| Clusterin (Clu) | UniProt ID P05371 | ASN42, ILE45, VAL49, VAL52, PHE194, PHE195, PHE243 ALA365, PHE368, PRO369, VAL371, ASP372, LYS375, PRO381 |
| TIMP metallopeptidase inhibitor 1(Timp1) | UniProt ID P30120 | PHE35, CYS36, LEU69, LYS70, GLY71, PHE72, ASP73, SER144, CYS147 |
| Vascular cell adhesion molecule 1 (Vcam1) | UniProt ID P29534 | PHE695, SER696, GLU698, LEU699, ALA701, LEU702, TYR703, ALA705, SER706, SER707, VAL709, ILE710, ILE713, ILE717, ARG721, TYR729, SER730, LEU731, VAL732, GLU733, ALA734, GLN735, LYS736 |
| Tissue inhibitor of metalloproteinases 2 (Timp2) | UniProt ID P30121 | ARG22, PRO23, ALA24, ASP25, ALA26, CYS27, SER28, CYS29, SER30, PRO31, VAL32, PRO34, ALA37, ASN40, ALA41, SER95, ALA96, ALA113, GLY114, LYS115, LEU126, CYS127, ASP128, PHE129, VAL131, THR135, LEU136, SER137, GLN140, ARG158, TRP174, TRP177, ILE183, ASN184, ALA188, LYS189 |
| Apolipoprotein E (Apoe) | UniProt ID P02650 | LEU8, LEU9, VAL10, PRO11, LEU12, LEU13, THR14, GLY15, CYS16, LEU17, ALA18, GLU19, GLY20, GLU21, LEU22, GLU23, VAL25, ASP26, GLN27, LEU28, PRO29, GLY30, GLN31, SER32, ASP33, GLN34, PRO35, TRP36, GLU37, GLN38, LEU40, ASN41, ARG42, TRP44, LEU47, ARG48, GLN62, SER63, SER64, THR67, GLN68, GLU69, THR71, VAL72, LEU73, GLU75, ASP76, THR79, GLU80, ALA83, TYR84, LYS86, GLU87, LEU88, GLU90, GLN91, GLY93, PRO94, ARG122, ASN123, GLY126, ARG129, ASN130, ASN133, THR134, ARG155, MET159, ALA162, ASP163, GLN166, LEU169, ALA170, LYS173, ALA174, GLY175, ALA176, GLN177, GLU178, ALA180 |
| Cystatin C | UniProt ID P14841 | LEU29, GLY31, ALA32, PRO33, GLN34, GLU35, ALA36, ASP37, ALA38, SER39, GLU40, GLU41, GLY42, VAL43, GLN44, ARG45, ALA46, LEU47, ASP48, PHE49, ALA50, VAL51, SER52, GLU53, TYR54, ASN55, LYS56, GLY57, SER58, ASN59, ASP60, ALA61, TYR62, HIS63, SER64, ARG65, ALA66, ILE67, GLN68, VAL69, VAL70, ARG71, ALA72, ARG73, LYS74, GLN75, LEU76, VAL77, ALA78, GLY79, ILE80, ASN81, TYR82, TYR83, LEU84, ASP85, VAL86, GLU87, MET88, GLY89, ARG90, THR91, THR92, CYS93, THR94, LYS95, SER96, GLN97, THR98, ASN99, LEU100, THR101, ASN102, CYS103, PRO104, PHE105, HIS106, ASP107, GLN108, PRO109, HIS110, LEU111, MET112, ARG113, LYS114, ALA115, LEU116, CYS117, SER118, PHE119, GLN120, ILE121, TYR122, SER123, VAL124, PRO125, TRP126, LYS127, GLY128, THR129, HIS130, THR131, LEU132, THR133, LYS134, SER135, SER136, CYS137, LYS138, ASN139, ALA140 |

**Table S4.** Binding energy values of gallic acid with the selected target proteins.

| **Ligand** | **Proteins** | **Binding energy** | **Hydrogen bonds** | | | | **Hydrophobic Interactions** | | | |
| --- | --- | --- | --- | --- | --- | --- | --- | --- | --- | --- |
|  |  |  | **No of H- Bonds** | **AA Residue** | **Ligand Atom** | **Distance (A)** | **No of Hydrophobic interactions** | **AA Residue** | **Type** | **Distance (A)** |
| Gallic acid | Timp1 | -4.97 | 5 | ARG111:HH21 | O5 | 2.40539 | 1 | ALA34 | Pi-Alkyl | 4.64825 |
|  |  |  |  | PHE124:HN | O1 | 1.97008 |  |  |  |  |
|  |  |  |  | SER38:OG | H17 | 2.10077 |  |  |  |  |
|  |  |  |  | CYS122:O | H16 | 2.10724 |  |  |  |  |
|  |  |  |  | ALA109:O | H15 | 2.00075 |  |  |  |  |
| Gallic acid | Igfbp7 | -4.87 | 6 | THR52:HG1 | O1 | 1.91077 | 1 | PRO82 | Pi-Alkyl | 5.13141 |
|  |  |  |  | GLY83:HN | O5 | 1.86775 |  |  |  |  |
|  |  |  |  | LYS113:HZ1 | O4 | 2.67134 |  |  |  |  |
|  |  |  |  | MET84:O | H18 | 1.97106 |  |  |  |  |
|  |  |  |  | CYS80:O | H17 | 2.26699 |  |  |  |  |
|  |  |  |  | CYS80:O | H15 | 2.13526 |  |  |  |  |
| Gallic acid | Kl | -4.81 | 4 | GLN398:HE22 | O3 | 2.75463 | 1 | ARG397 | Pi-Alkyl | 5.14016 |
|  |  |  |  | LYS445:HZ2 | O4 | 1.71651 |  |  |  |  |
|  |  |  |  | ALA55:O | H18 | 2.23562 |  |  |  |  |
|  |  |  |  | SER395:OG | H15 | 1.93889 |  |  |  |  |
| Gallic acid | Apoe | -4.76 | 6 | ARG152:HE | O5 | 2.81019 | 2 | ARG152 | Pi-Alkyl | 5.30279 |
|  |  |  |  | ARG152:HH22 | O5 | 2.01285 |  | LYS156 | Pi-Alkyl | 3.50882 |
|  |  |  |  | A:ARG155:HE | O1 | 2.22709 |  |  |  |  |
|  |  |  |  | MET159:SD | H17 | 2.66776 |  |  |  |  |
|  |  |  |  | ARG152:O | H16 | 2.02875 |  |  |  |  |
|  |  |  |  | MET159:SD | H15 | 2.3978 |  |  |  |  |
| Gallic acid | Timp2 | -4.58 | 5 | VAL32:HN | O5 | 1.63863 | NIL | | | |
|  |  |  |  | VAL32:O | H18 | 1.76033 |  |  |  |  |
|  |  |  |  | LEU126:O | H17 | 1.76678 |  |  |  |  |
|  |  |  |  | ASP128:O | H16 | 2.0177 |  |  |  |  |
|  |  |  |  | ASP128:O | H15 | 1.97782 |  |  |  |  |
| Gallic acid | Vcam1 | -4.53 | 6 | ARG721:HE | O4 | 1.80394 | NIL | | | |
|  |  |  |  | ARG721:HH21 | O5 | 2.00918 |  |  |  |  |
|  |  |  |  | LYS726:HN | O1 | 2.03858 |  |  |  |  |
|  |  |  |  | GLY727:HN | O2 | 2.16577 |  |  |  |  |
|  |  |  |  | ASN724:OD1 | H17 | 1.84663 |  |  |  |  |
|  |  |  |  | ASN724:OD1 | H15 | 2.18739 |  |  |  |  |
| Gallic acid | Cystatin C | -4.2 | 7 | HIS63:HE2 | O5 | 1.96513 | 3 | VAL51:CG1 | Pi-Sigma | 3.45502 |
|  |  |  |  | ARG65:HE | O4 | 1.91234 |  | ARG65 | Pi-Alkyl | 5.05706 |
|  |  |  |  | ARG65:HH21 | O4 | 3.00097 |  | ALA66 | Pi-Alkyl | 5.45877 |
|  |  |  |  | ALA66:HN | O2 | 2.77805 |  |  |  |  |
|  |  |  |  | THR101:O | H18 | 2.2299 |  |  |  |  |
|  |  |  |  | ASP48:O | H17 | 2.17436 |  |  |  |  |
|  |  |  |  | ASP48:OD1 | H15 | 2.15986 |  |  |  |  |
| Gallic acid | Tnf | -4.1 | 5 | ALA97:HN | O4 | 2.70768 | 2 | VAL96:CG2 | Pi-Sigma | 3.49742 |
|  |  |  |  | GLY226:HN | O5 | 1.77057 |  | ARG111 | Pi-Alkyl | 4.48469 |
|  |  |  |  | ALA97:O | H18 | 2.22992 |  |  |  |  |
|  |  |  |  | ALA112:O | H16 | 1.88244 |  |  |  |  |
|  |  |  |  | ALA112:O | H15 | 1.97046 |  |  |  |  |
| Gallic acid | Tlr4 | -3.88 | 4 | VAL714:O | H18 | 2.09182 | 2 | PHE710 | Pi-Pi T-shaped | 5.26375 |
|  |  |  |  | PHE710:O | H17 | 2.21602 |  | PRO712 | Pi-Alkyl | 5.45191 |
|  |  |  |  | HIS706:O | H16 | 2.13602 |  |  |  |  |
|  |  |  |  | HIS706:O | H15 | 1.93818 |  |  |  |  |
| Gallic acid | Clu | -3.76 | 4 | VAL49:O | H18 | 2.10581 | 2 | VAL49 | Pi | 5.25049 |
|  |  |  |  | HIS197:O | H17 | 1.78015 |  | LYS53 | Pi | 4.56889 |
|  |  |  |  | PHE195:O | H16 | 1.95129 |  |  |  |  |
|  |  |  |  | HIS197:O | H15 | 1.77423 |  |  |  |  |

**Table S5.** Binding energy values of alpha-tocopherol with the selected target proteins.

| **Ligand** | **Proteins** | **Binding energy** | **Hydrogen bonds** | | | | **Hydrophobic Interactions** | | | |
| --- | --- | --- | --- | --- | --- | --- | --- | --- | --- | --- |
|  |  |  | **No of H- Bonds** | **AA Residue** | **Ligand Atom** | **Distance (A)** | **No of Hydrophobic interactions** | **AA Residue** | **Type** | **Distance (A)** |
| Alpha-  Tocopherol | Kl | -8.14 | 1 | ASN514:OD1 | H81 | 2.25011 | 9 | TRP852 | Pi-Pi Stacked | 4.20494 |
|  |  |  |  |  |  |  |  | TRP852 | Pi-Pi Stacked | 4.33126 |
|  |  |  |  |  |  |  |  | LYS501 | Alkyl | 4.13603 |
|  |  |  |  |  |  |  |  | PRO742 | Alkyl | 4.68026 |
|  |  |  |  |  |  |  |  | LEU502 | Alkyl | 3.6674 |
|  |  |  |  |  |  |  |  | TYR434 | Pi-Alkyl | 5.25926 |
|  |  |  |  |  |  |  |  | PHE498 | Pi-Alkyl | 5.27644 |
|  |  |  |  |  |  |  |  | PHE508 | Pi-Alkyl | 4.18941 |
|  |  |  |  |  |  |  |  | PHE508 | Pi-Alkyl | 4.68622 |
| Alpha-  Tocopherol | Timp2 | -7.59 | 1 | ILE183:O | H81 | 1.8957 | 8 | A:VAL100 | Alkyl | 4.69226 |
|  |  |  |  |  |  |  |  | A:LEU126 | Alkyl | 4.99939 |
|  |  |  |  |  |  |  |  | A:ILE130 | Alkyl | 4.67464 |
|  |  |  |  |  |  |  |  | A:ILE183 | Alkyl | 5.31396 |
|  |  |  |  |  |  |  |  | VAL100 | Alkyl | 4.02965 |
|  |  |  |  |  |  |  |  | A:LEU126 | Alkyl | 5.0663 |
|  |  |  |  |  |  |  |  | A:PRO34 | Pi-Orbitals | 5.43945 |
|  |  |  |  |  |  |  |  | A:ILE183 | Pi-Orbitals | 5.08836 |
| Alpha-  Tocopherol | Clu | -7.54 | 1 | THR375:HG1 | O2 | 2.14779 | 8 | ALA372:CB | Pi-Sigma | 3.67225 |
|  |  |  |  |  |  |  |  | LEU371A,LA372:N | Amide-Pi Stacked | 4.81762 |
|  |  |  |  |  |  |  |  | VAL49 | Alkyl | 4.88282 |
|  |  |  |  |  |  |  |  | VAL49 | Alkyl | 4.91466 |
|  |  |  |  |  |  |  |  | VAL368 | Alkyl | 5.4143 |
|  |  |  |  |  |  |  |  | VAL49 | Alkyl | 4.95041 |
|  |  |  |  |  |  |  |  | PRO199 | Alkyl | 4.1963 |
|  |  |  |  |  |  |  |  | PHE194 | Pi-Alkyl | 4.5813 |
| Alpha-  tocopherol | Igfbp7 | -6.35 | 2 | VAL35:HN | O2 | 1.69393 | 5 | ALA55 | Alkyl | 4.37028 |
|  |  |  |  | PRO33 | H81 | 2.10697 |  | ALA55 | Alkyl | 4.69761 |
|  |  |  |  |  |  |  |  | VAL61 | Alkyl | 4.44578 |
|  |  |  |  |  |  |  |  | VAL61 | Alkyl | 4.58125 |
|  |  |  |  |  |  |  |  | CYS58 | Pi-Alkyl | 4.29506 |
| Alpha-  tocopherol | Tlr4 | -6.62 | 2 | HIS706:HD1 | O1 | 2.07754 | 5 | ILE716 | Alkyl | 5.19108 |
|  |  |  |  | PHE710 | H81 | 2.839 |  | HIS706 | Pi-Alkyl | 4.80479 |
|  |  |  |  |  |  |  |  | HIS706 | Pi-Alkyl | 3.75876 |
|  |  |  |  |  |  |  |  | PHE710 | Pi-Alkyl | 4.91334 |
|  |  |  |  |  |  |  |  | TRP744 | Pi-Alkyl | 4.36876 |
| Alpha-  Tocopherol | Vcam1 | -5.62 | 3 | LYS692:HN | O2 | 2.14492 | 3 | TYR694 | Pi-Alkyl | 5.20061 |
|  |  |  |  | PHE695:HN | O1 | 2.1492 |  | TYR694 | Pi-Alkyl | 3.78956 |
|  |  |  |  | ASN690 | H81 | 1.71044 |  | PHE695 | Pi-Alkyl | 5.11037 |
| Alpha-  tocopherol | Timp1 | -5.48 | 2 | ASN37:OD1  LYS70 | H81  HZ1 | 2.07928  3.19979 | 3 | LYS70 | Alkyl | 5.10202 |
|  |  |  |  |  |  |  |  | LYS70 | Alkyl | 4.5181 |
|  |  |  |  |  |  |  |  | LYS70 | Alkyl | 4.72744 |
| Alpha-  Tocopherol | Cystatin C | -5.45 | 2 | THR98:O | H18 | 2.07113 | 6 | LYS56 | Alkyl | 5.40402 |
|  |  |  |  | ASN99:CA | O2 | 3.00555 |  | LYS95 | Alkyl | 3.65866 |
|  |  |  |  |  |  |  |  | VAL51:C17 | Alkyl | 4.38064 |
|  |  |  |  |  |  |  |  | HIS63 | Pi-Alkyl | 4.08249 |
|  |  |  |  |  |  |  |  | HIS63:C17 | Pi-Alkyl | 4.52491 |
|  |  |  |  |  |  |  |  | LEU100 | Pi-Alkyl | 4.96705 |
| Alpha-  Tocopherol | Tnf | -5.23 | 2 | ASN113:HD21 | O1 | 2.31038 | 8 | VAL92:C31 | Alkyl | 4.4474 |
|  |  |  |  | ARG111:CD | O2 | 3.65755 |  | ILE233:C31 | Alkyl | 3.71832 |
|  |  |  |  |  |  |  |  | HIS94 | Pi-Alkyl | 5.47649 |
|  |  |  |  |  |  |  |  | HIS94 | Pi-Alkyl | 3.64783 |
|  |  |  |  |  |  |  |  | TYR138 | Pi-Alkyl | 5.14022 |
|  |  |  |  |  |  |  |  | VAL96 | Pi-Alkyl | 5.38178 |
|  |  |  |  |  |  |  |  | ARG111 | Pi-Alkyl | 4.88671 |
|  |  |  |  |  |  |  |  | ALA112 | Pi-Alkyl | 4.92823 |
| Alpha-  Tocopherol | Apoe | -4.05 | 2 | GLN56:HE22 | O2 | 2.40604 | 7 | TRP49 | Pi-Sigma | 3.40766 |
|  |  |  |  | GLU60:OE1 | H81 | 2.23177 |  | TRP49 | Pi-Pi Stacked | 4.14724 |
|  |  |  |  |  |  |  |  | TRP49 | Pi-Pi Stacked | 4.69536 |
|  |  |  |  |  |  |  |  | ARG42 | Alkyl | 4.55382 |
|  |  |  |  |  |  |  |  | LEU73 | Alkyl | 4.58481 |
|  |  |  |  |  |  |  |  | TYR46 | Pi-Alkyl | 5.38588 |
|  |  |  |  |  |  |  |  | TYR46 | Pi-Alkyl | 4.6636 |
